# A single-domain antibody detects and neutralises toxic Aβ_42_ oligomers in the Alzheimer’s disease CSF

**DOI:** 10.1186/s13195-023-01361-z

**Published:** 2024-01-18

**Authors:** Alessandra Bigi, Liliana Napolitano, Devkee M. Vadukul, Fabrizio Chiti, Cristina Cecchi, Francesco A. Aprile, Roberta Cascella

**Affiliations:** 1 Department of Experimental and Clinical Biomedical Sciences, Section of Biochemistry, University of Florence, Florence, Italy; 2 Department of Chemistry, Molecular Sciences Research Hub, Imperial College London, London, UK; 3 Institute of Chemical Biology, Molecular Sciences Research Hub, Imperial College London, London, UK

**Keywords:** Nanobodies, Conformation-sensitive antibodies, Amyloid β peptide, Protein misfolding, Early diagnosis, Biofluids, Aβ_42_-targeting therapy, Neurodegenerative diseases

## Abstract

**Background:**

Amyloid-β_42_ (Aβ_42_) aggregation consists of a complex chain of nucleation events producing soluble oligomeric intermediates, which are considered the major neurotoxic agents in Alzheimer’s disease (AD). Cerebral lesions in the brain of AD patients start to develop 20 years before symptom onset; however, no preventive strategies, effective treatments, or specific and sensitive diagnostic tests to identify people with early-stage AD are currently available. In addition, the isolation and characterisation of neurotoxic Aβ_42_ oligomers are particularly difficult because of their transient and heterogeneous nature. To overcome this challenge, a rationally designed method generated a single-domain antibody (sdAb), named DesAb-O, targeting Aβ_42_ oligomers.

**Methods:**

We investigated the ability of DesAb-O to selectively detect preformed Aβ_42_ oligomers both in vitro and in cultured neuronal cells, by using dot-blot, ELISA immunoassay and super-resolution STED microscopy, and to counteract the toxicity induced by the oligomers, monitoring their interaction with neuronal membrane and the resulting mitochondrial impairment. We then applied this approach to CSF samples (CSFs) from AD patients as compared to age-matched control subjects.

**Results:**

DesAb-O was found to selectively detect synthetic Aβ_42_ oligomers both in vitro and in cultured cells, and to neutralise their associated neuronal dysfunction. DesAb-O can also identify Aβ_42_ oligomers present in the CSFs of AD patients with respect to healthy individuals, and completely prevent cell dysfunction induced by the administration of CSFs to neuronal cells.

**Conclusions:**

Taken together, our data indicate a promising method for the improvement of an early diagnosis of AD and for the generation of novel therapeutic approaches based on sdAbs for the treatment of AD and other devastating neurodegenerative conditions.

**Supplementary Information:**

The online version contains supplementary material available at 10.1186/s13195-023-01361-z.

## Background

Alzheimer’s disease (AD) is the most prevalent neurodegenerative disorder affecting ca. 60–70% of 55 million people worldwide suffering with some form of dementia (Alzheimer’s association, 2023). It is characterised by the presence of extracellular amyloid-β (Aβ) plaques and intracellular tau neurofibrillary tangles in specific vulnerable populations of neurons, thus leading to their death [1–3]. In the last decades, small oligomers of the 42-residue form of Aβ (Aβ_42_), formed early during the aggregation process or released from mature fibrils, have acquired increasing importance as primary toxic species in AD pathogenesis [4–7]. Indeed, elevated oligomer levels in the brain are associated with pathology [8] and correlate better with the degree of dementia as compared to mature fibrils [9]. Moreover, in some transgenic mouse models overexpressing mutant amyloid precursor protein (APP), synaptic alteration and cognitive impairment precede amyloid plaque formation, but occur after Aβ levels start to rise steadily [10, 11].

In the past few years, considerable effort has been made to identify the structural determinants of oligomer neurotoxicity, including oligomer size and hydrophobic exposure [12, 13], that are responsible for their ability to trigger several toxic pathways, ultimately leading to neuronal death [5, 13, 14]. Notably, soluble Aβ oligomers have been recently identified within the cerebrospinal fluid (CSF) of mild cognitive impaired (MCI) and AD [15–17] patients. In particular, conformation-sensitive antibodies (Abs) directed against well-defined forms of Aβ_42_ oligomers, have detected oligomers in AD brains that were absent in age-matched healthy individuals [18–21]. The aggregate distribution varies in terms of structure, size and shape but also toxicity along AD progression [17], so that an efficient method to distinguish and quantify these species could serve diagnostic and therapeutic purposes. Indeed, the lack of specific and sensitive diagnostic tests to identify people with early-stage AD to be included in clinical trials is among the main reasons for many notable trial failures, considering that cerebral lesions occur 20 years before symptom onset [22–25].

Over the past 25 years, several monoclonal Abs (mAbs) have been engineered to bind and eliminate Aβ [26] from the brain of AD patients and are currently under investigation in clinical trials. Although some Abs, such as Aducanumab (Aduhelm™), initially appeared to be able to eliminate parenchymal amyloid [27], they have so far failed to change cognition in subjects with MCI and AD [9]. More recently, in 2023, the FDA approved another mAb, Lecanemab (Leqembi™), which slows down cognitive decline in AD [28]. Both mAbs are recommended for the early stages of AD but, unfortunately, they can only slow down AD decline without stopping it completely or reverting it [28–30].

The capture of small soluble protein aggregates to reduce their toxicity could be an important therapeutic strategy for AD. In this context, nanobodies or single-domain Abs (sdAbs) could constitute a real breakthrough for the treatment of AD [31], but also for early diagnosis of AD. sdAbs, firstly discovered in 1993, are recombinant, antigen-specific, variable fragments of camelid heavy chain-only Abs (VHH) [32]. As classical Abs, they retain high target specificity and affinity, but their small size makes them highly stable, soluble, able to access clefts and hidden epitopes and to be functionally expressed as intrabodies [33]. Moreover, their low immunogenic potential and inherent toxicity make them great tools for basic research and potential candidates for both diagnostic and therapeutic applications [31, 33].

Previously published works [34, 35] demonstrated that targeting the region 29 to 36 of Aβ_42_ with rationally designed Abs, also called DesAbs [36, 37], can inhibit the peptide’s secondary nucleation. Among these DesAbs, DesAb-O was found to preferentially bind to Aβ_42_ oligomers rather than its monomeric and fibrillar forms, as this region is likely to be solvent-exposed when the peptide is oligomeric, before becoming buried in amyloid fibrils [35].

In this study, we investigated the ability of DesAb-O to selectively detect preformed Aβ_42_ oligomers both in vitro and in cultured cells and to counteract their neurotoxicity, taking advantage of commercially available conformation-sensitive Abs, as controls. By using dot-blot, ELISA immunoassay and stimulated emission depletion (STED) microscopy, we demonstrated the high ability of DesAb-O to identify Aβ_42_ oligomers rather than monomers and fibrils. Moreover, DesAb-O was found to significantly inhibit oligomer binding to neuronal membranes, restoring mitochondrial functionality. We then applied this approach to CSF samples (CSFs) from AD patients as compared to healthy individuals. We demonstrated the presence of well-resolved Aβ_42_ oligomeric species in the CSF of AD patients, clearly detected by DesAb-O. Moreover, the administration of AD CSFs to cultured neuronal cells caused detrimental effects that were completely abolished by the pre-incubation with DesAb-O.

These findings strongly suggest that the use of sdAbs to detect soluble toxic species in biofluids offers a promising way for the improvement of an early diagnosis of AD. Our study also reveals a powerful ability of DesAb-O to prevent Aβ_42_ neurotoxicity, contributing to the generation of novel therapeutic approaches based on sdAbs for AD and other neurodegenerative diseases.

## Methods

### *Preparation of Aβ*_*42*_* aggregates*

Aβ_42_ conformers were prepared as previously reported [38–40]. Briefly, the lyophilised peptide (Bachem) was dissolved in 100% hexafluoro-2-isopropanol (HFIP) to 1 mM and the solvent was then evaporated under nitrogen. To obtain Aβ_42_ oligomers, the peptide was resuspended in 50 mM NaOH at 1 mg/ml and diluted in PBS to a final concentration of 25 μM. Then, the sample was centrifuged at 22,000* g* for 30 min, the pellet discarded and the supernatant incubated at 25 °C without agitation for 1 day to obtain A + oligomers or for 4 days to obtain A − oligomers [40]. F1 were obtained, with the same procedure, at a final concentration of 50 μM after 1 day of incubation [40]. Amyloid β-derived diffusible ligands (ADDLs) were obtained by dissolving an aliquot of the peptide in anhydrous dimethyl sulfoxide (DMSO) to 5 mM and then diluting in ice-cold F-12 medium to a final concentration of 100 μM. This solution was incubated at 4 °C for 1 day and then centrifuged at 14,000 × *g* for 10 min [38]. Finally, F2 were prepared by dissolving the peptide in DMSO to 5 mM and then diluting it in 10 mM HCl to a final concentration of 100 μM. The sample was incubated at 37 °C without agitation for 1 day [39].

#### CSF samples

CSF samples (CSFs) from human aged controls (*n* = 4) or AD patients (*n* = 9) were obtained from BioIVT. Each CSF was received in 0.5–1 ml aliquot and stored at − 80 °C. Samples were centrifuged at 4000* g* for 10 min at 4 °C, obtaining a pale pellet that was separated from the supernatant. The supernatant was then analysed; protein concentration in these samples was determined by the Bradford colorimetric method [41].

#### DesAb-O and DesAb18–24 purification

DesAb-O and DesAb18–24 were purified as previously described [34]. Briefly, DesAb-O and DesAb18–24 were expressed in *E.Coli* Origami™ (DE3) pLysS cells (Merck Millipore), and grown for at least 15 h at 30 °C in Overnight Express Instant TB Medium (Merck Millipore) supplemented with 100 μg/ml ampicillin. Cells were collected by centrifugation, resuspended in PBS (8 mM Na_2_HPO_4_, 15 mM KH_2_PO_4_, 137 mM NaCL, and 3 mM KCl, pH 7.3) with an EDTA-Free Complete Protease Inhibitor Cocktail tablet (Roche). Cells were lysed by sonication and cellular debris was removed by centrifugation. The supernatant was applied to a HisTrap HP 5 ml column (Cytiva) that has been pre-equilibrated with PBS supplemented with 15 mM imidazole. The column was then washed with several column volumes of PBS supplemented with 15 mM imidazole after which the Ab was eluted in PBS with 300 mM imidazole. The sample was then dialysed against PBS overnight at 4 °C to remove imidazole, after which it was applied to a HiLoad Superdex 75 16/600 pg (Cytiva) column for size exclusion chromatography. Protein concentration was determined by measuring the absorbance at 280 nm and using the molecular coefficient of DesAb-O or DesAb18–24.

#### Dot-blot analysis

Dot-blot analysis was performed by spotting 2.0 μl (corresponding to 0.1 μg) of each Aβ_42_ conformer onto a nitrocellulose membrane. After 30 min blocking (1.0% bovine serum albumin, BSA, in TBS/TWEEN 0.1%), Aβ_42_ species were probed with 2 μM DesAb-O or with 1:15.000 diluted human monoclonal anti-ADDLs (19.3) Ab (Creative Biolabs), or with 1:1000 diluted rabbit polyclonal anti-oligomer (A11) Ab (Thermo Fisher Scientific), or with 1:1000 diluted rabbit polyclonal anti-amyloid fibrils (OC) Ab (Sigma-Aldrich), or with 1:1000 diluted mouse monoclonal anti-Aβ (6E10) Ab (Biolegend Way) and then with 1:2000 diluted goat anti-6X His tag (Abcam), or goat anti-human (Sigma-Aldrich), or goat anti-rabbit (Abcam) or rabbit anti-mouse (Abcam) horseradish peroxidase (HRP) conjugated secondary Abs. In another set of experiments, decreasing quantities (0.1, 0.05, 0.0025, 0.001 and 0.005 μg) of Aβ_42_ aggregates were spotted onto the nitrocellulose membrane, and then probed with DesAb-O and 19.3 Abs, as described above. The immunolabelled dots were detected using a SuperSignalWest Dura (Pierce) ImageQuant™ TL software (GE Healthcare UK Limited version 8.2).

In a set of experiments, sandwich dot-blot was performed. Briefly, 2 µl of 6E10 and DesAb-O Abs (0.01 mg/ml and 10 µM, respectively) were spotted onto nitrocellulose membranes. After 20 min, the blots were blocked in TBS-Tween-20 0.2% and 2.5% BSA IgG free for 40 min. The membranes were incubated with different Aβ_42_ species at 0.01 mg/ml (monomeric Aβ_42_, A + oligomers, F1) or with the CSFs of AD patients and control subjects at 0.1 mg/ml. Then, the membranes were probed with 1:1000 diluted 6E10 Ab overnight at 4 °C under constant shaking. The following day, the membranes were washed three times in TBS-Tween-20 0.2% and incubated with 1:3000 diluted rabbit anti-mouse HRP-conjugated secondary Ab for 1 h. After three additional washes, the immunolabelled dots were detected as reported above.

#### ELISA assay

For indirect ELISA assay, increasing concentrations (0, 1, 5, and 10 µM, monomer equivalents) of each Aβ_42_ conformer, prepared as reported above, were immobilised on a 96-well Maxisorp ELISA plate (Nunc) under quiescent conditions for 1 h at room temperature (RT). The plate was then washed three times with 20 mM Tris, pH 7.4, and 100 mM NaCl and incubated in 20 mM Tris, pH 7.4, 100 mM NaCl, and 5% BSA under constant shaking overnight at 4 °C. The day after the plate was washed six times with 20 mM Tris, pH 7.4, and 100 mM NaCl and then incubated with 40 μL solutions of 2.0 μM DesAb-O, or with 1:20,000 diluted 19.3 Ab, or with 1:8000 diluted 6E10 Ab, under constant shaking for 1 h at RT. The plate was then washed six times with 20 mM Tris, pH 7.4, and 100 mM NaCl and incubated with 1:2000 diluted goat anti-6X His tag, 1:5000 diluted goat anti-human and 1:4000 rabbit anti-mouse HRP-conjugated secondary Abs, in 20 mM Tris, pH 7.4, 100 mM NaCl, and 5% BSA under shaking for 1 h at RT. Finally, the plate was washed three times with 20 mM Tris, pH 7.4, and 100 mM NaCl, then twice with 20 mM Tris, pH 7.4, 100 mM NaCl, and 0.02% Tween-20, and again three times with 20 mM Tris, pH 7.4, and 100 mM NaCl. Finally, the amount of bound Abs was quantified by using 1-Step Ultra TMB-ELISA Substrate Solution (Thermo Fisher Scientific), according to the manufacturer’s instructions, and the reaction was stopped by adding 40 μl of H_2_SO_4_. Then, the absorbance was measured at 450 nm by means of a CLARIOstar plate reader (BMG Labtech).

For sandwich ELISA assay, 1 µM DesAb-O or 0.5 µM DesAb18–24 Abs were immobilised on a 96-well Maxisorp ELISA plate under quiescent conditions for 1 h at RT. After three washes in PBS, the plate was blocked with 5% BSA IgG free overnight at 4 °C under constant shaking. The day after, the plate was washed six times in PBS and different Aβ_42_ species (M, A + oligomers, F1 at decreasing concentrations (4500, 2250, 450, 45, 4.5 and 2.25 pg/ml for DesAb-O and 4500∙10^3^ pg/ml, 4500 pg/ml and 4.5 pg/ml for DesAb18–24) and the CSFs of AD patients (*n* = 9) and control subjects (*n* = 4) at 0.25 mg/ml were loaded into the plate overnight at 4 °C under constant shaking. In the DesAb-O plate, we loaded 4500 pg/ml of monomeric αSynuclein (αSyn) as negative control. The following day after six additional washes in PBS, the plate was incubated with 1:4000 6E10 Ab for 2 h at RT with no shaking, while αSyn was incubated with 1:4000 diluted mouse monoclonal anti-αSyn (211) Ab (Santa Cruz Biotechnology). The plate was washed six times in PBS-Tween-20 0.2% and incubated with 1:5000 rabbit anti-mouse HRP- conjugated secondary Ab for 1 h at RT. The plate was then washed six additional times in PBS-Tween-20 0.2% and the amount of Aβ_42_ species bound was quantified as reported above.

#### Cell cultures

Authenticated human SH-SY5Y neuroblastoma cells were purchased from A.T.C.C. and cultured in Dulbecco’s modified Eagle’s medium (DMEM), F-12 Ham with 25 mM 4-(2-Hydroxyethyl) piperazine-1-ethanesulfonic acid (HEPES) and NaHCO_3_ (1:1) supplemented with 10% foetal bovine serum (FBS), 1.0 mM glutamine and 1.0% penicillin and streptomycin solution (Sigma-Aldrich). Cells were maintained in a 5.0% CO_2_ humidified atmosphere at 37° C and grown until 80% confluence for a maximum of 20 passages, and tested to ensure that they were free form mycoplasma contamination [42]. Primary rat cortical neurons (Thermo Fisher Scientific) were plated in 12-well plate containing glass coverslips and maintained in neuronal basal plus medium (Thermo Fisher Scientific) supplemented with GlutaMAX (Gibco, Thermo Fisher Scientific) at the concentration of 0.5 mM and 2% (v/v) B-27 serum-free complement (Gibco, Thermo Fisher Scientific), in a 5% CO_2_ humidified atmosphere at 37 °C. Every 2 days the medium was partially replaced with fresh one. The experiments were performed 14 days after plating [43].

#### STED microscopy

Aβ_42_ assemblies were added to the culture medium of SH-SY5Y cells seeded on glass coverslips for 1 h at 3 μM (monomer equivalents). After incubation, the cells were washed with PBS, the plasma membranes were counterstained with 0.01 mg/ml tetramethylrhodamine-conjugated wheat germ agglutinin (WGA;Thermo Fisher Scientific) [44] for 15 min at 37 °C and cells were then fixed with 2.0% (w/v) paraformaldehyde for 10 min at RT. After washing with PBS, the plasma membranes were permeabilised with a 3.0% (v/v) glycerol solution for 10 min at RT. Aβ_42_ species were then detected with 4 μM DesAb-O and 1:800 diluted FITC anti-6X tag secondary Abs (Abcam), or with 1:250 diluted human monoclonal (19.3) anti-oligomer Ab and 1:1000 diluted Alexa Fluor 488-conjugated anti-human secondary Ab (Thermo Fisher Scientific), or with 1:400 diluted A11 Ab, or with 1:800 diluted OC Ab, or with 1:400 diluted 6E10 Ab and 1:500 diluted Alexa Fluor 514-conjugated anti-rabbit or anti-mouse secondary Abs (Thermo Fisher Scientific). STED xyz images (i.e., *z*-stacks acquired along 3 directions: *x*, *y*, and *z* axes) were acquired as previously reported [44]. In a set of experiments, SH-SY5Y cells seeded on glass coverslips were experienced for 24 h with ADDLs at 3.0 μM (monomer equivalents) or CSFs from AD patients or controls (*n* = 4 for both AD and control CSFs) diluted 1:1 with cellular medium. In another set of experiments, primary rat cortical neurons were treated with CSFs from AD patients or controls (*n* = 4 for both AD and control CSFs) diluted 1:1 with cellular medium. After the incubation, the cells were counterstained and analysed by STED microscopy as reported above. In another set of experiments, A + oligomers, F1 and a mixture containing both Aβ_42_ species (1:1 molar ratio) were incubated on a glass coverslip at 25 µM in PBS without cells while the CSFs (*n* = 9 and *n* = 4 for AD and controls, respectively) were spotted at a concentration of 0.5 mg/ml. After 30 min of incubation, the samples were blocked in Casein 1X with TBS-Tween-20 0.2% for 30 min. Once washed with TBS-Tween-20 0.2%, Aβ_42_ species and the CSFs were detected with 1:800 diluted 6E10, 2.0 μM DesAb-O or 4 µM DesAb18–24 Ab for 1 h and then with 1:500 diluted Alexa Fluor 514-conjugated anti-mouse secondary Abs or with 1:500 FITC anti-6X tag secondary Abs. The acquisition was performed as reported above.

#### Confocal microscopy analysis of Aβ_42_ aggregates bound to neuronal membranes

SH-SY5Y cells were seeded on glass coverslips and then treated for 15 min with A + oligomers or ADDLs at a concentration of 3.0 μM (monomer equivalents), in the absence or presence of a pre-incubation with increasing molar ratios (1:0.1, 1:0.25, 1:0.5 and 1:1, monomer equivalents) between Abs and Aβ_42_ species. After incubation, the cells were washed with PBS and the plasma membranes were counterstained for 15 min with 5.0 μg mL^−1^ Alexa Fluor 633-conjugated WGA (Thermo Fisher Scientific) [6]. Cells were fixed in 2.0% (w/v) paraformaldehyde for 10 min at RT and Aβ_42_ assemblies were detected with 1:800 diluted 6E10 Ab and then 1:1000 diluted Alexa Fluor 488-conjugated anti-mouse secondary Ab (Thermo Fisher Scientific). To detect only the oligomers bound to the cell surface, the cellular membrane was not permeabilised, thus preventing Ab internalisation. Fluorescence emission was detected after double excitation at 633 and 488 nm by a TCS SP8 scanning confocal microscopy system (Leica Microsystems), as previously described [45]). The degree of colocalisation of Aβ_42_ aggregates with the cell membranes was estimated in regions of interest in 30–32 cells, via the use of ImageJ software (NIH) and JACOP plugin (http://rsb.info.nih.gov) (Rasband WR).

#### MTT reduction assay

The cytotoxicity of the different Aβ_42_ aggregates was assessed in SH-SY5Y cells seeded in 96-well plates by the 3-(4,5-dimethylthiazol-2-yl)-2,5-diphenyltetrazolium bromide (MTT) assay [46, 47]. Briefly, Aβ_42_ species (monomer, A + and A − oligomers, ADDLs, F1 and F2) at a concentration of 3.0 μM (monomer equivalents) were added to the culture medium of SH-SY5Y cells for 24 h following or not a pre-incubation under shaking with equimolar concentrations of DesAb-O, 19.3, A11 or OC Abs. After treatment, the culture medium was removed, cells were washed with PBS and the MTT assay was assessed as previously reported [7]. Cell viability was expressed as the percentage of MTT reduction in treated cells as compared to those untreated (taken as 100%), or to those treated with Aβ_42_ species in the absence of Abs.

In a set of experiments, CSFs (*n* = 4 for AD as well as controls) were added to the culture medium of SH-SY5Y cells for 24 h following a 1 h pre-incubation in the absence or presence of DesAb-O at 3 µM at 37 °C under shaking conditions. Cells treated with ADDLs at 1 µM (monomer equivalents) were used as positive control. The analysis was performed as reported above.

#### Measurement of cytosolic free Ca^2+^ levels

The intracellular calcium levels were measured in SH-SY5Y cells as previously described [7, 48]. SH-SY5Y cells were treated for 5 h with the CSFs (*n* = 4 for AD as well for controls) in the absence or presence a 1 h pre-incubation with DesAb-O at 3 µM under shaking. At the end of the treatment, the cells were washed in PBS and loaded with 4.5 µM Fluo-4 AM (Thermo Fisher Scientific) for 10 min and cytosolic Ca^2+^ levels were detected after excitation at 488 nm by the TCS SP8 scanning confocal microscopy system as previously reported [7, 44]. Cells treated with ADDLs at 1 µM (monomer equivalents) were used as positive control. Images were then analysed using the ImageJ software, and the fluorescence intensities were expressed as the percentage of that measured in untreated cells, taken as 100%.

#### Measurements of calcein leakage

The intracellular calcein levels were measured in SH-SY5Y cells as previously described [44]. Briefly, the cells were washed in PBS and loaded with 0.5 µM Calcein-AM (Thermo Fisher Scientific) for 20 min at 37 °C. Then, they were washed with PBS and treated for 5 h with CSFs (*n* = 4 for both AD and control CSFs) in the absence or presence of a 1 h pre-incubation with DesAb-O at 3 µM under shaking. Cells treated with ADDLs at 1 µM (monomer equivalents) were used as positive control. After fixation in 2.0% buffered paraformaldehyde for 10 min at RT, fluorescence emission was detected after excitation at 488 nm by the TCS SP8 scanning confocal microscopy system as previously reported [44]. Images were then analysed as reported above.

#### Statistical analysis

Data were expressed as means ± standard deviation (S.D.), or as means ± standard error of mean (S.E.M). Comparisons between the different groups were performed by using the Student *t* test or the one-way ANOVA followed by Bonferroni’s multiple-comparison test (GraphPad Prism 5.0 software). *P* values lower than 0.05, 0.01 and 0.001 were considered to be statistically significant (*), highly statistically significant (**) and very highly statistically significant (***), respectively.

## Results

### DesAb-O selectively detects synthetic Aβ_42_ oligomers in vitro

We firstly evaluated the specificity of DesAb-O against different Aβ_42_ conformers by dot-blot analysis. Aβ_42_ conformers were assembled in vitro according to well-defined protocols. Thus, toxic A + and nontoxic A − oligomers [40], toxic Aβ_42_ ADDLs [38] and two types of fibrils, namely F1 [40] and F2 [39] were formed and their identities were confirmed by routinely analysing them by atomic force microscopy (AFM) and dot-blots using their respective conformation-sensitive Abs, as previously reported [49]. The various Aβ_42_ species were then deposited (2 μl, corresponding to 0.1 μg) onto a nitrocellulose membrane and detected with DesAb-O and, as a control, with the 19.3 Ab, specific for amyloid β-derived diffusible ligands (ADDLs) [16], A11 Ab, recognising toxic oligomers from various proteins, but not their monomeric or fibrillar conformations [18], and OC Ab, specifically raised against fibrillar aggregates [20].

**Fig. 1:**
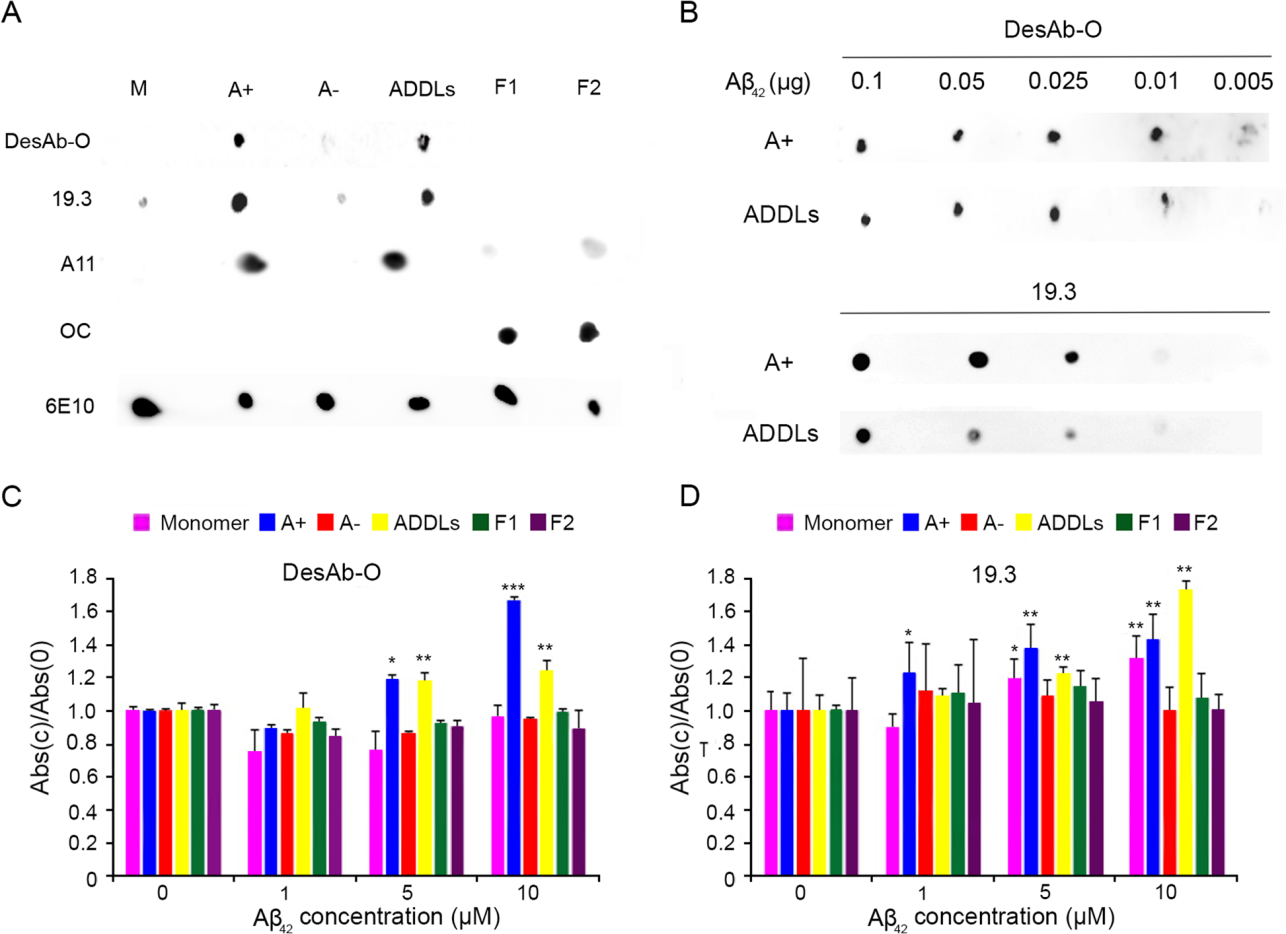
DesAb-O selectively detects synthetic Aβ_42_ oligomers in vitro. **A**,**B** Dot-blot analysis of Aβ_42_ species. **A** Samples of monomeric Aβ_42_ (M), oligomeric (A + , A − and ADDLs) and two types of fibrillar species (F1 from [40] and F2 from [39]) were deposited (2 μl/spot, corresponding to 0.1 μg) onto a nitrocellulose membrane and detected with the indicated Abs. **B** Samples of A + oligomers and ADDLs at various amounts (0.25, 0.10, 0.05, 0.025, 0.01, 0.005 μg) were probed with DesAb-O (top) and 19.3 (bottom) Abs. **C**,**D** ELISA measurements taken at increasing concentration of Aβ_42_ species using DesAb-O (**C**) and 19.3 (**D**) Abs. Data were normalised for the corresponding average value at concentration 0 μM. Experimental errors are S.D. (*n* = 3). Samples were analysed by Student* t* test relative to 0 μM (*P < 0.05, **P < 0.01 and ***P < 0.001)

DesAb-O was found to selectively target toxic A + oligomers and ADDLs, with a minor cross-reaction with nontoxic A − oligomers (Fig. 1A). As expected, each of the abovementioned control Abs bound to their targeted conformers according to previous reports [16, 18, 20, 40, 45]. Proper loading of each Aβ_42_ species was confirmed by the sequence-specific 6E10 Ab which targets the N-terminus of Aβ_42_ [18, 20, 50, 51], that bound all the analysed Aβ_42_ species, independently of their aggregation state (Fig. 1A).

We then assessed the sensitivity of DesAb-O by evaluating its ability to probe decreasing amounts of Aβ_42_ conformers (0.10, 0.05, 0.025, 0.01 and 0.005 μg), previously deposited onto a nitrocellulose membrane (Fig. 1B). DesAb-O was found to detect A + oligomers and ADDLs down to 0.01 μg, which appeared more sensitive when compared to the control 19.3 Ab (Fig. 1B).

The reactivity of DesAb-O against Aβ_42_ species was also quantified by performing an indirect ELISA assay. Briefly, we coated the wells of the ELISA plates with increasing concentrations of Aβ_42_ conformers (0, 1, 5 and 10 μM, monomer equivalents). We then incubated the plates with DesAb-O and subsequently with the appropriate secondary Abs. Our results showed that DesAb-O clearly recognised toxic A + oligomers and ADDLs at 5 and 10 µM, with a specificity that increased with aggregate concentration (Fig. 1C), revealing no affinity for the monomeric or fibrillar forms even at high concentrations (Fig. 1C)_._ The 19.3 Ab was also assessed for a relative comparison, revealing high affinity for A + oligomers and ADDLs, even at 1 µM for the former species, but showing a minor specificity for Aβ_42_ monomers (Fig. 1D). As expected, the 6E10 Ab was found to detect all the analysed Aβ_42_ conformers in a dose-dependent manner (Fig. S1), as the Aβ_42_ N-terminus is solvent-exposed regardless of the aggregated state of the peptide [17]. Overall, dot-blot and ELISA results demonstrate the ability of DesAb-O to selectively discriminate Aβ_42_ conformers, in agreement with previously established work [34, 35]. Moreover, the affinity of DesAb-O against Aβ_42_ oligomers and its selectivity for the oligomeric species with respect to the monomers and fibrils do not seem to be any worse than those observed with commercially available Abs, providing the platform for further applications.

### *DesAb-O detects synthetic Aβ*_*42*_* oligomers bound to neuronal membrane and internalised into the cytosol*

**Fig. 2:**
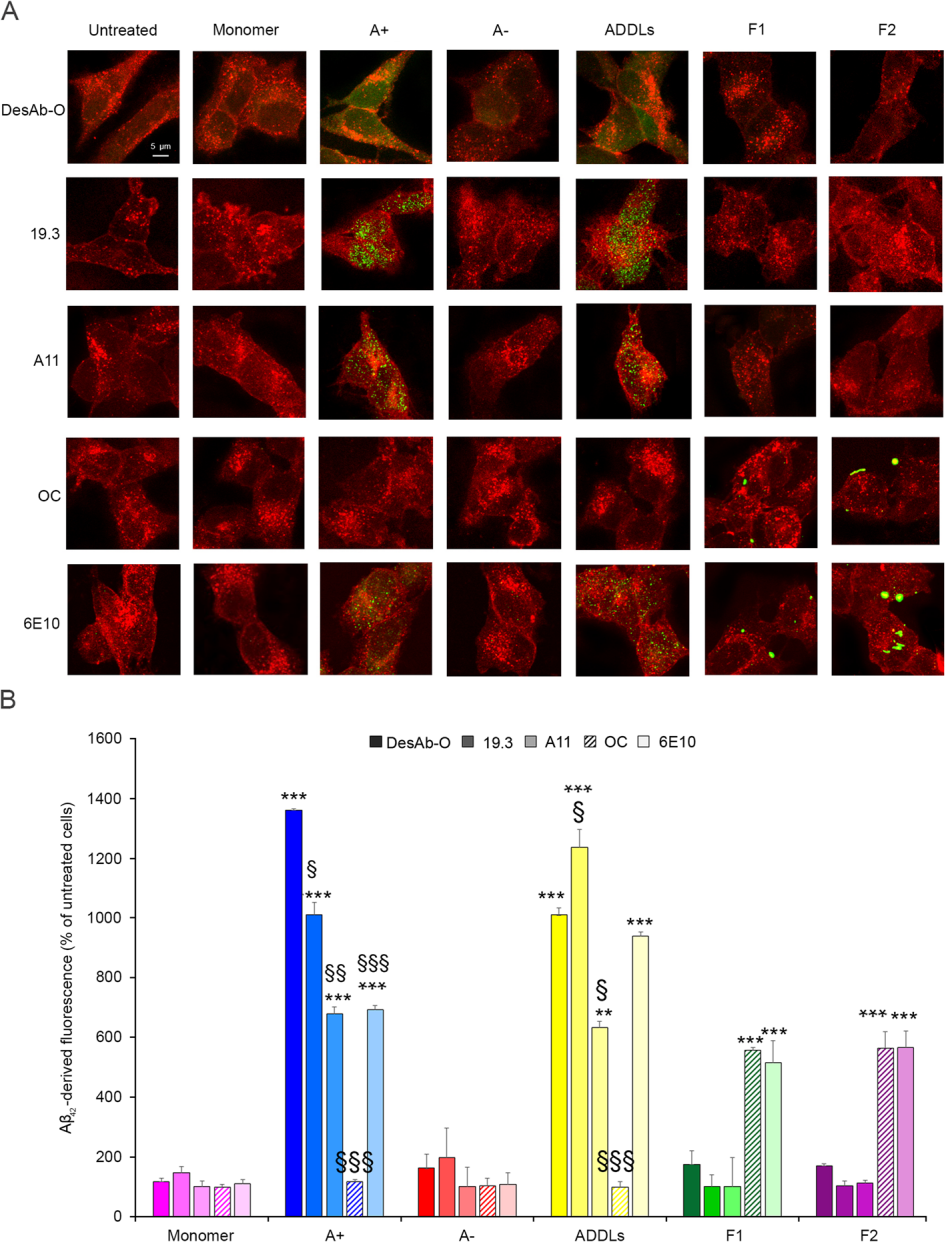
DesAb-O detects synthetic Aβ_42_ oligomers interacting with neuronal cells.** A** Representative STED microscopy images of SH-SY5Y cells treated with the indicated Aβ_42_ species at 3.0 μM (monomer equivalents) for 1 h. Red and green fluorescence indicates respectively the cell membranes and the Aβ_42_ species, detected with the indicated Abs. **B** The histograms represent the results of a semi-quantitative analysis of the green fluorescent signal. Experimental errors are S.E.M. (*n* = 3). Samples were analysed by Student* t* test relative to untreated cells (**P < 0.01, and ***P < 0.001), or to cells treated with the same Aβ_42_ species and detected with DesAb-O (§*P* < 0.05, §§*P* < 0.01, §§§*P* < 0.001). 200–250 cells were analysed per condition

To analyse whether or not the ability of DesAb-O to selectively detect Aβ_42_ oligomers in vitro is also reflected in cultured cells, human neuroblastoma SH-SY5Y cells were exposed for 1 h to different types of Aβ_42_ species at 3.0 μM (monomer equivalents) and then the plasma membrane (red channel) and Aβ_42_ aggregates (green channel) were counterstained and analysed by the super-resolution STED microscope [44] (Fig. 2). DesAb-O was found to detect toxic A + oligomers and ADDLs (Fig. 2A), showing an increase of the green fluorescent signal by 1362 ± 46% and 1010 ± 45%, respectively, with respect to the untreated cells, taken as 100% (Fig. 2B). In particular, by analysing different optical sections (apical, median and basal planes to the coverslip), DesAb-O can identify these oligomers bound to the neuronal membranes and penetrating into the cells (Fig. S2). Similar results were obtained with the 19.3 Ab (1010 ± 99% and 1235 ± 39%) and to a lower extent with the A11 one (680 ± 66% and 633 ± 97%) (Fig. 2A,B). The OC Ab specifically recognised both types of fibrillar conformers (F1 and F2), which appeared predominantly bound to the plasma membranes, and the green fluorescent signal increased by 557 ± 55% and 562 ± 57% for the former type of fibril [40] and the latter type of fibril [39], respectively (Fig. 2A,B). As expected, the sequence-specific 6E10 Ab detected both A + oligomers and ADDLs and F1 and F2 fibrils on neuronal cells (Fig. 2A,B). None of the Abs detected nontoxic A − oligomers that evoked a very low and undetectable fluorescent signal because they are known to weakly interact with neuronal membranes [40, 45, 49].

Overall, in our experimental conditions, DesAb-O selectively discriminated toxic Aβ_42_ oligomers with respect to monomeric and fibrillar forms of the peptide, at least similarly to the other commercially available conformation-sensitive Abs, suggesting a very promising potential for the detection of harmful Aβ_42_ species in biological fluids.

#### DesAb-O inhibits the interaction of Aβ_42_ oligomers with neuronal membranes preventing mitochondrial dysfunction

**Fig. 3:**
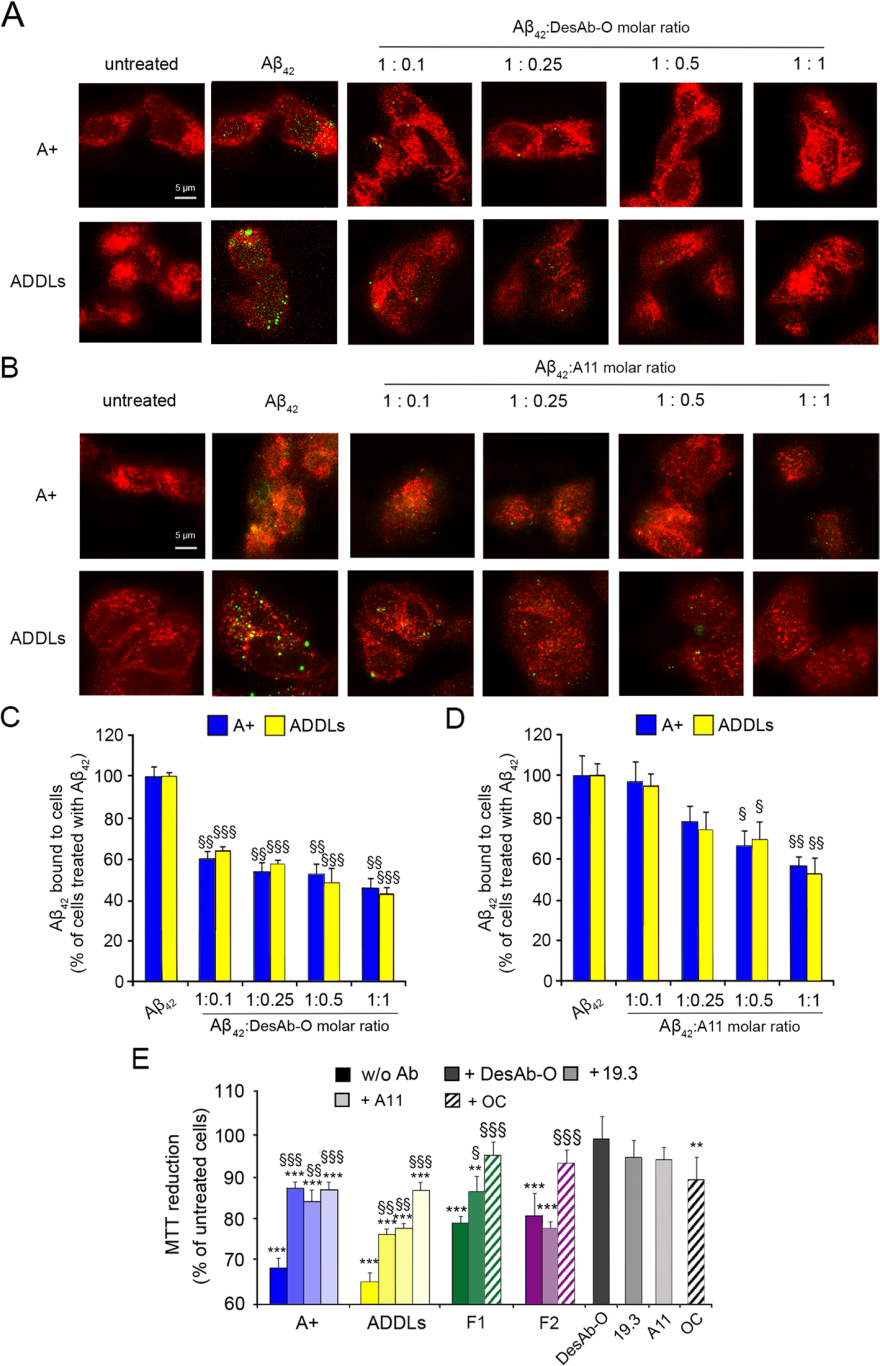
DesAb-O inhibits the binding of Aβ_42_ oligomers to the neuronal membrane preventing their induced mitochondrial dysfunction. **A**,**B** Representative confocal microscopy images of SH-SY5Y cells treated with 3.0 μM (monomer equivalents) A + oligomers and ADDLs following 1 h pre-incubation in the absence or presence of DesAb-O (**A**) or A11 (**B**) Abs at the indicated Aβ_42_:Abs molar ratios, where molar ratios refer to monomer equivalents. Red and green fluorescence indicates the cell membranes and Aβ_42_ oligomers detected with the 6E10 Ab. **C**,**D** Degree of membrane binding of A + oligomers and ADDLs measured following incubation under the conditions represented in panels A and B, determined as reported in the *Methods* section. **E** MTT reduction in SH-SY5Y cells treated for 24 h with the indicated Aβ_42_ aggregates at a concentration of 3.0 μM (monomer equivalents) following a 1 h pre-incubation in the absence or presence of the indicated Abs. Abs alone were also tested as a control. Experimental errors are S.E.M. (*n* = 4). Samples were analysed by one-way ANOVA followed by Bonferroni’s multiple-comparison test relative to untreated cells (*P < 0.05, **P < 0.01, and ***P < 0.001), or to cells treated with the same Aβ_42_ species without any Ab (§*P* < 0.05, §§*P* < 0.01, §§§*P* < 0.001). 200–250 (**A–D**) and 250,000–300,000 (**E**) cells were analysed per condition

We further evaluated whether DesAb-O was also able to capture Aβ_42_ oligomers, preventing their detrimental effects on neuronal cells. To this purpose, A + oligomers and ADDLs were pre-incubated for 1 h with DesAb-O at increasing molar ratios between oligomers and Ab (from 1:0.1 to 1:1), and these solutions were then added to the cell culture medium of SH-SY5Y cells for 15 min. Unlike previous experiments, the oligomers were added to cultured cells only after pre-incubation with DesAb-O. To detect only the oligomers bound to the cell surface, the cellular membrane was not permeabilised at this stage, thus preventing antibody internalisation. The binding affinity of the aggregates for cellular membranes was assessed by confocal microscopy using the 6E10 Ab as a probe. Our results showed a strong colocalisation of Aβ_42_ A + oligomers and ADDLs with the neuronal membranes in the absence of the pre-incubation with Ab (Fig. 3A), confirming previously reported data [45, 52, 53]. Notably, the binding of both types of Aβ_42_ aggregates was significantly reduced in the presence of DesAb-O up to 1:0.1 molar ratio (by 40 ± 3% and 36 ± 2%, respectively) (Fig. 3A,C). The same analysis was performed with the A11 Ab, which was found to prevent the interaction of the oligomers with the membrane only at 1:1 molar ratio (by 44 ± 5% and 48 ± 7%, respectively) (Fig. 3B,D). These results again suggested a great affinity of DesAb-O for the oligomers, at least equal to that of a traditional conformation-sensitive Ab.

We then evaluated whether DesAb-O was also able to prevent the neurotoxic effects evoked by Aβ_42_ aggregates, by analysing their mitochondrial status with the MTT reduction test. Aβ_42_ species (3.0 μM, monomer equivalents) were incubated in the absence or presence of an equimolar concentration of DesAb-O for 1 h, and then these solutions were added to the culture medium of SH-SY5Y cells for 24 h. Our results showed that A + oligomers and ADDLs significantly reduced (by 31 ± 3% and 35 ± 3%, respectively) the mitochondrial activity of SH-SY5Y cells as compared to untreated cells, taken as 100% (Fig. 3E), as previously shown [6, 18, 40, 45, 48]. Both types of fibrils were also found to be significantly toxic, even if to a lesser extent with respect to the oligomeric species (the reduction of cell viability was 21 ± 2% and 17 ± 2% for F1 and F2, respectively, as reported in Fig. 3E), confirming previous data [39, 40, 45]. When A + oligomers and ADDLs were pre-incubated for 1 h with DesAb-O, we observed a significant improvement of mitochondrial function (by 18 ± 4% and 11 ± 3%, respectively, with respect to cells treated with the same species in the absence of DesAb-O), whereas the fibril-induced neurotoxicity was not affected by DesAb-O (Fig. 3E), confirming again its high specificity in the targeting of Aβ_42_ oligomeric conformations. The same analysis was performed with the 19.3 and A11 Abs that were found to significantly prevent the cytotoxicity induced by A + oligomers (increase of MTT reduction by 15 ± 5% and 18 ± 4%, respectively) and ADDLs (by 13 ± 3% and 22 ± 3%, respectively), whereas the OC Ab markedly suppressed the cytotoxicity of fibrillar conformations (by 16 ± 4% and 16 ± 5%, respectively), as already shown [45]. Of note, DesAb-O did not affect neuronal viability when added alone to the cell culture medium, thus making it an excellent tool for future tentative therapeutic applications.

#### DesAb-O detects Aβ_42_ oligomers in the CSF of AD patients in vitro

**Fig. 4:**
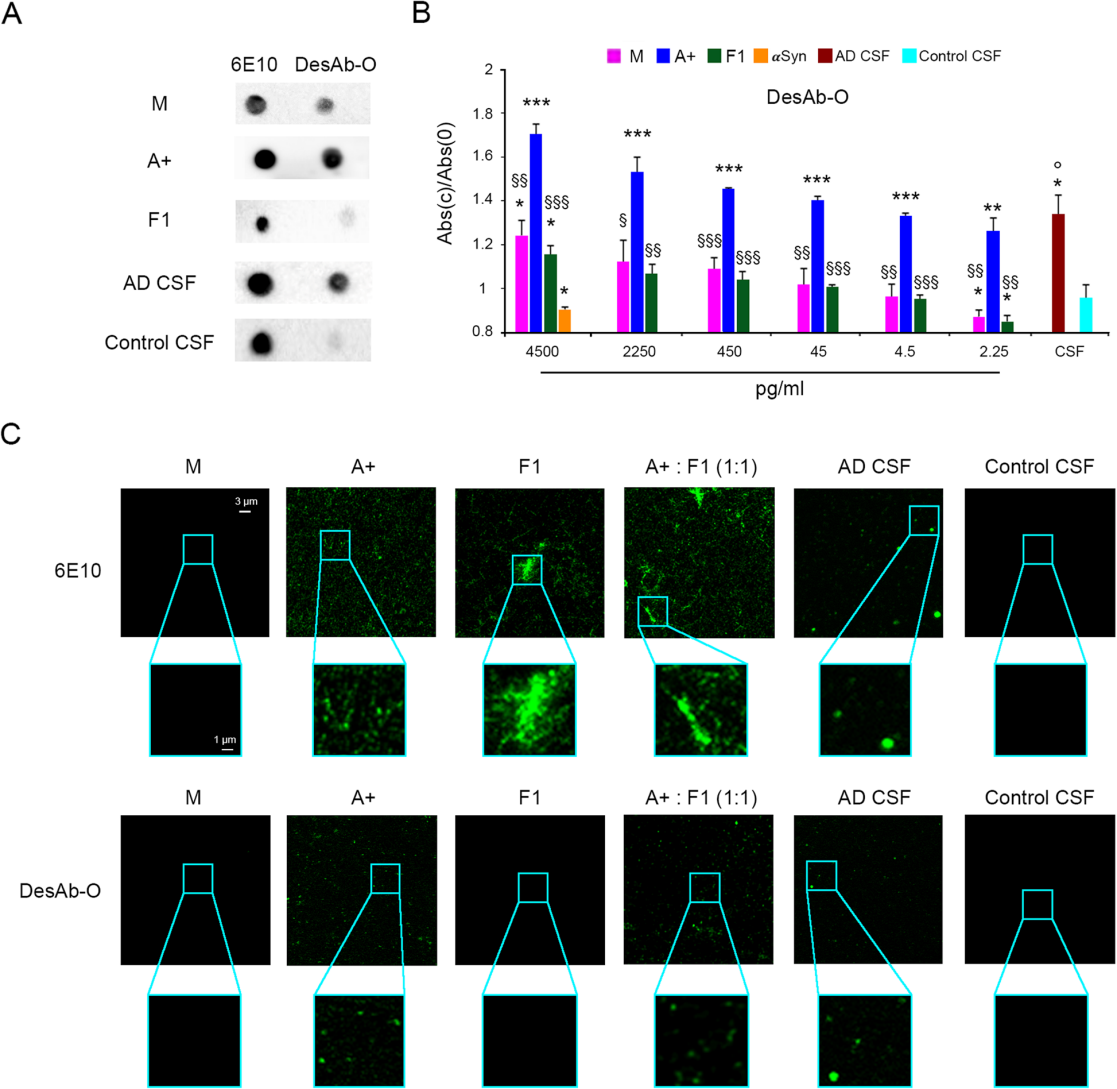
DesAb-O detects Aβ_42_ oligomers in the CSFs of AD patients in vitro.** A** Representative **s**andwich dot-blot analysis of Aβ_42_ species and CSFs. The capture Abs, 6E10 and DesAb-O were spotted onto nitrocellulose membranes (2 µl corresponding to 0.01 mg/ml and 10 µM). The membranes were incubated with solutions containing different Aβ_42_ species (monomeric Aβ_42_ (M), A + oligomers, fibrils (F1) at 0.01 mg/ml, the CSF from a representative AD patient (*n* = 9) and that from a representative control subject (*n* = 4) at 0.1 mg/ml. Finally, the membranes were probed with the detection 6E10 Ab. **B** Indirect sandwich ELISA. 0.25 mg/ml of CSFs from AD patients and control subjects were adsorbed and quantified by using DesAb-O at 1 µM. Standard curve was obtained with decreasing concentration of Aβ_42_ species formed in vitro. αSyn monomeric protein was used as a negative control. Data were normalised for the corresponding average value at concentration 0 pg/ml. Experimental errors are S.E.M (*n* = 4 for synthetic samples and control CSFs and *n* = 9 for AD CSFs). Samples were analysed by Student *t* test relative to 0 pg/ml (*P < 0.05, **P < 0.01, ***P < 0.001) or to A + (§*P* < 0.05, §§*P* < 0.01, §§§*P* < 0.001) or to control CSF (°*P* < 0.05). **C** Representative STED images showing Aβ_42_ species (M, A + , F1, a mixture containing A + and F1 at 1:1 molar ratio) and CSFs collected from AD patients and controls spotted in a glass coverslip at 25 µM and 0.5 mg/ml, respectively (*n* = 4 for synthetic samples and control CSFs and *n* = 9 for AD CSFs). The green fluorescent signals arise from the staining with 6E10 and DesAb-O Abs. Higher magnifications of the Aβ_42_ species are shown in the boxed areas

Considering the encouraging data obtained with DesAb-O both in vitro and in cultured cells, we then assessed its ability to identify Aβ_42_ species that are present in the CSF of AD patients with respect to the CSF of age-matched control subjects. We performed a series of proof-of-concept experiments on a small set of clinical samples of CSF (*n* = 9 from AD and *n* = 4 from controls) to explore whether our assays could detect differences between the two groups. We first performed a sandwich dot-blot analysis by spotting 2 µl of the capture Abs 6E10 and DesAb-O (corresponding to 0.01 mg/ml and 10 µM, respectively) onto a nitrocellulose membrane that was then incubated with Aβ_42_ species at 2 µM or CSF from AD patients and controls at 0.1 mg/ml. The membranes were then revealed with the 6E10 Ab. This approach, which is different from the classical dot-blot employed in Fig. 1A,B, is useful in a context in which the amount of oligomers we expected to have in the CSF of AD patients was very low. The 6E10 Ab, as expected, showed positive signal with all samples, including the CSFs (Fig. 4A). Despite the improved sensitivity in the recognition of oligomeric species, that gave rise to a high signal (Fig. 4A), DesAb-O was also found to generate a slight cross-reaction with monomers and to a minor extent with fibrils. Interestingly, an intense spot was observed following the incubation of DesAb-O with AD CSFs and only a weak signal with the control CSFs (Fig. 4A).

To further demonstrate the capability of DesAb-O to identify Aβ_42_ oligomers in the CSF of AD patients with respect to control individuals, we performed an indirect sandwich ELISA assay, again to improve specificity and sensitivity. Briefly, we coated the ELISA plate with 1 µM DesAb-O and incubated with decreasing concentrations (4500, 2250, 450, 45, 4.5 and 2.25 pg/ml) of Aβ_42_ species (Aβ_42_ monomer (M), A + oligomers, and F1, the CSFs from AD patients (*n* = 9) and that from control subjects (*n* = 4) at the concentration of 0.25 mg/ml. The plates were then probed with 6E10 as capture Ab. We found that DesAb-O significantly recognised A + oligomers (Fig. 4B, blue bars) down to 2.25 pg/ml with respect to the monomeric and fibrillar forms of Aβ_42_ (Fig. 4B, magenta and green bars, respectively)_,_ showing a lower affinity for these forms only at the highest concentration. As a control, we used monomeric αSyn, which was not recognised by DesAb-O, demonstrating again its high specificity for Aβ_42_ (Fig. 4B, orange bar). Notably, DesAb-O generated a high signal with the CSFs of AD patients (Fig. 4B, dark red bar) that appeared significantly different from that obtained with those of control subjects (Fig. 4B, cyan bar).

The ability of DesAb-O to detect Aβ_42_ oligomers in the CSFs of AD patients was also exploited by super-resolution STED microscopy [44]. Aβ_42_ species (25 µM, monomer equivalents) and CSFs from AD patients and controls (0.5 mg/ml) were deposited on a glass coverslip and labelled with 6E10 and DesAb-O Abs. As expected, the 6E10 Ab clearly recognised both preformed A + oligomers and fibrils, even in a 1:1 mixture between oligomeric and fibrillar species (Fig. 4C). In particular, A + oligomers exhibited green fluorescent punctae, which appeared to be small and globular at the very high magnifications allowed by STED microscopy, whereas F1 appeared fibrillar in morphology (Fig. 4C, bottom box magnifications). Monomeric Aβ_42_ is difficult to detect (Fig. 4C), in agreement with the results obtained in a cellular context (Fig. 2A,B). For this reason, the 6E10 Ab did not detect Aβ species in control CSF AD samples, whereas it did with AD CSFs, recognising green fluorescent punctae, globular in shape, some of which apparently larger than oligomeric species (Fig. 4C, bottom box magnifications). In contrast, DesAb-O clearly recognises A + oligomers rather than fibrils, revealing the presence of small globular green fluorescent punctae in the solutions containing the 1:1 mixture (Fig. 4C, bottom box magnifications). We then evaluated the CSFs, observing that DesAb-O can selectively detect small, globular and round species compatible with Aβ_42_ oligomers in the CSFs of AD patients, displaying no signal in the control ones (Fig. 4C, bottom box magnifications).

To validate our experimental approach, we tested another sdAb named DesAb18–24, which has been rationally designed to target Aβ_42_ fibrils, specifically the region VFFAEDVG [17, 34]. We thus performed an indirect sandwich ELISA assay by coating the wells with 0.5 µM DesAb18–24 and then performing the test as described above. Our results showed that DesAb18–24 clearly recognises Aβ_42_ fibrils down to 4.5 pg/ml, with low affinity for monomeric Aβ_42_ down to 4500 pg/ml and no binding for A + oligomers, except at the highest concentration (Fig. S3A). Notably, DesAb18–24 was found to generate a higher absorbance value from the control CSFs with respect to that observed from AD patients, because of the high cross-reaction with the monomers (Fig. S3B), confirming the difference in terms of total Aβ_42_ between patients and controls reported in literature. The specificity of DesAb18–24 was also evaluated by STED microscopy, showing a clear detection of preformed Aβ_42_ fibrils, without any signal for both CSFs as the monomeric protein is difficult to reveal in this experimental condition (Fig. S3B).

#### DesAb-O detects Aβ_42_ oligomers present in AD CSFs upon their interaction with neuronal cells

**Fig. 5:**
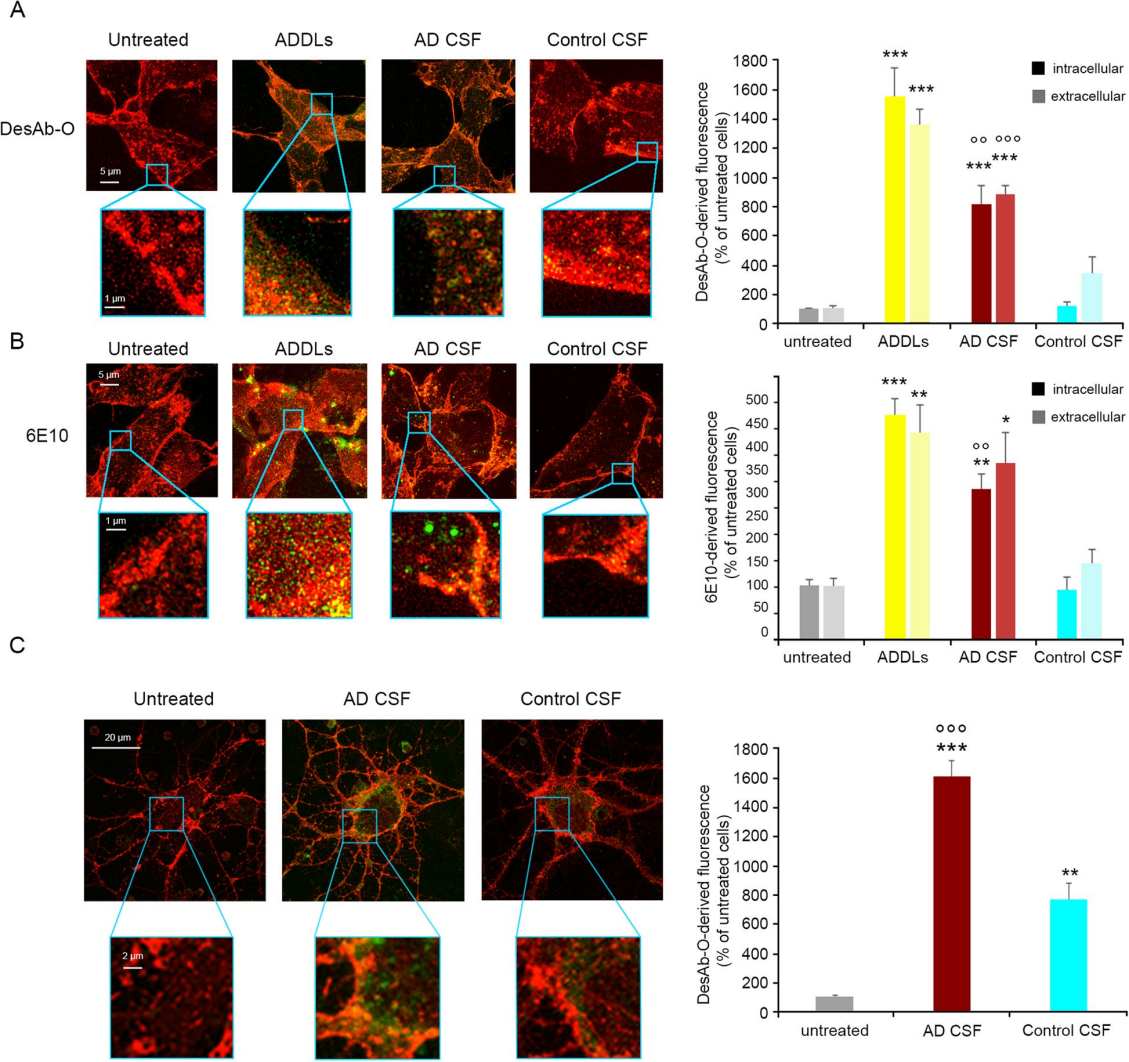
DesAb-O detects Aβ_42_ oligomers present in the CSFs of AD patients upon their interaction with neuronal cells. **A**,**B** Representative STED microscopy images of SH-SY5Y cells treated with ADDLs at 3.0 μM (monomer equivalents) or CSFs from AD patients and age-matched controls diluted 1:1 with the extracellular medium, for 5 h. Red and green fluorescence indicates the cell membranes and the Aβ_42_ species detected with DesAb-O (**A**) and 6E10 (**B**), respectively. Higher magnifications of the Aβ_42_ species are shown in the boxed areas. The histograms represent the results of a semi-quantitative analysis of the green fluorescent signal. **C** Representative STED microscopy images of primary rat cortical neurons treated with AD and control CSFs, as reported in **A** and **B**. Red and green fluorescence indicates respectively the cell membranes and the Aβ_42_ species detected with DesAb-O. Higher magnifications of the Aβ_42_ species are shown in the boxed areas. Experimental errors are S.E.M. (*n* = 4 for synthetic samples and control CSFs and *n* = 9 for AD CSFs). 200–250 cells were analysed per condition. Samples were analysed by the Student *t* test relative to untreated cells (*P < 0.05, **P < 0.01 and ***P < 0.001), or to cells treated with control CSFs (°°*P* < 0.01 and °°°*P* < 0.001)

To further evaluate the ability of DesAb-O to target Aβ_42_ oligomers present in the CSFs of AD patients, we applied high-resolution STED microscopy to SH-SY5Y cells exposed to ADDLs, or to the CSFs of AD patients and age-matched control subjects (without pre-incubation with Abs). Following the administration for 5 h of ADDLs at 3.0 μM (monomer equivalents) or CSFs diluted 1:1 with the extracellular medium, the Aβ_42_ aggregates (green channel) were counterstained with DesAb-O or 6E10 Abs and the cell membrane (red channel) with WGA (Fig. 5A,B). Cells exposed to ADDLs exhibited green fluorescent punctae, which appeared to be small and globular at the very high magnifications allowed by STED microscopy (Fig. 5A,B, bottom image magnification). The DesAb-O derived green fluorescent signals were consistent with the results obtained with the 6E10 Ab. In particular, the semi-quantitative analysis revealed that the oligomeric species are localised both intracellularly and extracellularly. Notably, DesAb-O can recognise a number of small and globular intracellular and extracellular Aβ_42_ species in the CSFs of AD patients, with a morphology that resembles that of the oligomeric species (Fig. 5A, bottom image magnification). In addition to the small oligomeric species, the 6E10 Ab can also recognise larger aggregates in the CSFs of AD patients that, at high magnification, appeared round in morphology (Fig. 5B, bottom image magnification). In contrast, cells treated with the CSFs of control subjects and counterstained with both DesAb-O and 6E10 showed the presence of few Aβ_42_ aggregates outside the cells or attached to the membrane, which probably represent nontoxic oligomers that are not able to permeabilise the cell membrane or low amounts of toxic oligomers that do not manifest their toxicity due to their small quantity (Fig. 5AB, bottom image magnification). Similar results were obtained in primary rat cortical neurons exposed to AD and control CSFs and labelled with DesAb-O (Fig. 5C).

#### DesAb-O prevents neuronal dysfunction induced by the CSFs of AD patients

**Fig. 6:**
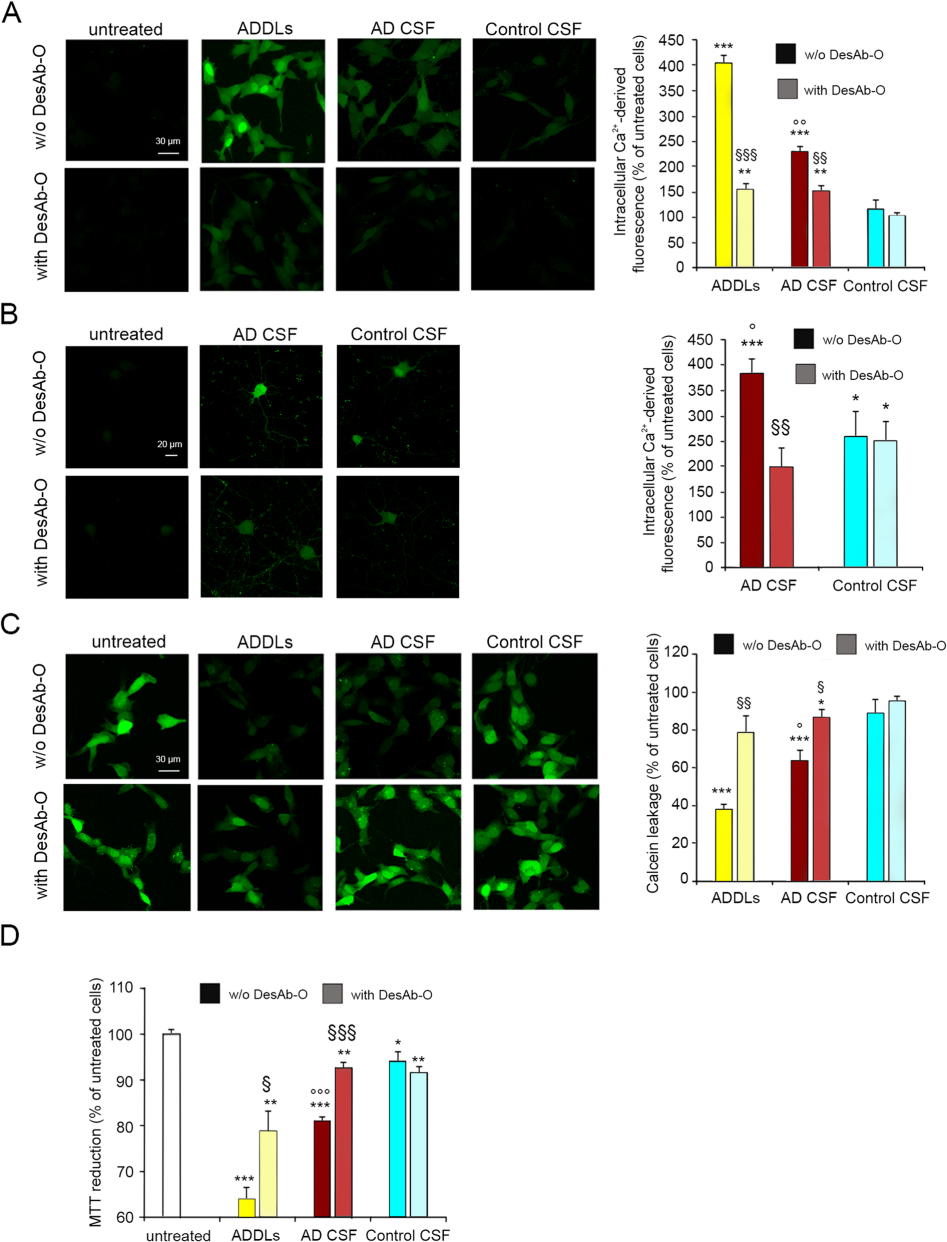
DesAb-O prevents neuronal dysfunction induced by the CSFs of AD patients. **A** Intracellular Ca^2+^-derived fluorescence in SH-SY5Y cells treated for 5 h with ADDLs at 1 µM (monomer equivalents), or with CSFs from AD patients and age-matched control subjects (*n* = 4), diluted 1:1 with the extracellular medium, following 1 h pre-incubation in the absence or presence of DesAb-O at 3 µM. **B** Intracellular Ca^2+^-derived fluorescence in primary rat cortical neurons treated for 2.5 h with CSFs from AD patients and control subjects (*n* = 4), diluted 1:1 with the extracellular medium, following 1 h pre-incubation in the absence or presence of DesAb-O at 3 µM. **C** Intracellular calcein-derived fluorescence in SH-SY5Y cells treated for 5 h with ADDLs at 1 µM (monomer equivalents), or with CSFs from AD patients and control subjects (*n* = 4), diluted 1:1 with the extracellular medium, following 1 h pre-incubation in the absence or presence of DesAb-O at 3 µM. In all panels, untreated cells are also shown. **D** MTT reduction in SH-SY5Y cells treated for 24 h with ADDLs at 1 µM (monomer equivalents), or with CSFs from AD patients and control subjects (*n* = 4), diluted 1:1 with the extracellular medium, following 1 h pre-incubation in the absence or presence of DesAb-O at 3 µM. Experimental errors are S.E.M. Samples were analysed by Student *t* test relative to untreated cells (*P < 0.05, **P < 0.01 and ***P < 0.001) or to cells treated with samples without DesAb-O (§*P* < 0.05, §§*P* < 0.01 and §§§*P* < 0.001) or to cells treated with control CSFs (°*P* < 0.05 and °°*P* < 0.01). 200–250 (**A**,**C**), 80–150 (**B**) and 250,000–300,000 (**D**) cells were analysed per condition

We finally evaluated whether DesAb-O can also neutralise the cytotoxicity of oligomers present in the CSFs of AD patients. A relatively high volume (ml of sample) is required to perform these experiments, so they were performed on 4 AD and 4 age-matched control CSFs. We first monitored the dysregulation of cytosolic Ca^2+^ homeostasis, which is an early upstream event evoked by extracellular Aβ_42_ oligomers both in cultured neuronal cells and in relevant mouse AD models, where Ca^2+^ ions flow from the extracellular space to the cytosol [7, 43, 54–57]. SH-SY5Y cells were treated for 5 h with the CSFs from AD patients and control subjects diluted 1:1 with the cell culture medium, following or not a 1 h pre-incubation with DesAb-O at 3 µM. The CSFs of AD patients caused a significant influx of Ca^2+^ ions (by 230 ± 11%) relative to untreated cells (Fig. 6A), whereas the CSFs of control subjects generated only a slight and non-significant alteration of Ca^2+^ homeostasis (Fig. 6A). A 1 h pre-incubation with DesAb-O significantly reduced the effect induced by the CSFs of AD patients (by 77 ± 20%), without affecting that observed in cells treated with the control ones (Fig. 6A). As a positive control, 1 µM (monomer equivalents) ADDLs were found to generate an extensive Ca^2+^ influx (by 405 ± 15%, Fig. 6A) that markedly decreased (by 250 ± 26%) following a 1 h pre-incubation with 3 µM DesAb-O. Similar results were obtained in primary rat cortical neurons exposed for 2.5 h to the CSFs of AD patients and control subjects following or not a 1 h pre-incubation with DesAb-O at 3 µM (Fig. 6B). These results confirm the high specificity of DesAb-O in the targeting of neurotoxic Aβ_42_ conformers present in AD CSFs.

The protective effect of DesAb-O was also observed from the analysis of the alteration of membrane permeability induced by Aβ_42_ oligomers, monitoring the release of the fluorescent probe calcein-acetoxymethyl (AM), previously loaded into the cells [48]. SH-SY5Y cells pre-loaded with calcein-AM were treated with the CSFs of AD patients and controls diluted 1:1 with the extracellular medium for 5 h following or not a 1 h pre-incubation with DesAb-O at 3 µM. Unlike control CSFs, the AD ones caused a significant permeabilisation of the cellular membrane relative to untreated cells, albeit to a lesser extent than ADDLs, used as a positive control (Fig. 6C). Interestingly, DesAb-O significantly prevented the membrane permeabilisation induced by AD CSFs causing an increase of intracellular calcein-derived fluorescence (by 23 ± 9%), although to a minor extent with respect to that observed when pre-incubated with ADDLs (by 41 ± 11%) (Fig. 6C).

We also carried out an MTT reduction test following the administration of CSFs diluted 1:1 with the extracellular medium for 24 h. Unlike the control CSFs, those derived from AD patients caused a modest reduction of the mitochondrial activity of SH-SY5Y cells (by 19 ± 1%) that was reduced by the 1 h pre-incubation with 3 µM DesAb-O, evident as an improvement of mitochondrial function of 11 ± 2% (Fig. 6D). ADDLs caused a significant reduction of cell viability (by 36 ± 4%) that was prevented by DesAb-O, resulting in an increase of mitochondrial function of 15 ± 7% (Fig. 6D), confirming previous results shown in Fig. 3E.

Overall, these results confirmed the selective ability of DesAb-O to bind and neutralise both in vitro synthesised and patient-derived Aβ_42_ oligomers, representing a promising tool for a future diagnostic, therapeutic and prognostic application in AD.

## Discussion

Soluble oligomers of Aβ_42_ have been widely associated with neuronal dysfunction and synaptic loss in AD [5, 13, 14]. Conformational Abs raised by different investigators against independently generated Aβ oligomers have detected these species in AD brains unlike age-matched healthy individuals [18–21]. In the last few years, it has also been demonstrated that Aβ oligomers are present in the CSF of MCI and AD cases, representing a key biomarker candidate [15–17]. Consistent efforts have been made to develop robust assays to characterise and quantify oligomers, discriminating them from the monomeric form [15–17, 58]. In all the ELISA-based methods, a significant overlap in the total mass of Aβ oligomers between patients and controls has been observed, although a small increase in the oligomeric mass has been reported for some cohorts [16, 59]. Recently, highly sensitive biophysical methods have indirectly identified toxic oligomers in the CSF of MCI patients rather than in AD cases, even in a small number of samples [17, 60, 61]. In addition, multiple aggregated forms of Aβ_42_, varying in terms of structure, size, shape and neurotoxicity, have been reported along AD progression [17]. However, due to the lack of suitable sensitive methods, the detection, quantification and isolation of these soluble neurotoxic species from biological fluids remain difficult, because of their heterogeneity, transient nature and very low concentration.

Nanobodies or sdAbs have been recently proposed as promising tools for basic research and potential candidates for diagnostic and therapeutic applications in a range of pathological conditions, thanks to their high target specificity and affinity, as well as low immunogenic potential [33, 62]. In particular, the rational design of sdAbs that selectively target specific Aβ conformers neutralising their neurotoxicity has a great potential of diagnostic and therapeutic value for AD [35, 62–66]. A dozen of sdAbs have shown such potential value for AD in vitro [67], and two of them, namely R3VQ and A2, have reached in vivo imaging as they bind brain Aβ deposits and tau inclusions, respectively [68].

In this work, we examined the possible role of a sdAb, named DesAb-O, targeting a conformational epitope formed by residues 29–36 of Aβ_42_ and exposed by the oligomeric species [35], to selectively detect and neutralise Aβ_42_ oligomers both from synthetic origin and present in AD CSFs. We first characterised its ability to detect a range of pathologically relevant, highly stable and well-characterised Aβ_42_ aggregates (A + and A − oligomers, ADDLs, and two types of fibrils) [38–40], taking advantage of a panel of commercially available conformation-sensitive Abs as controls. The immunoassay analysis revealed a high affinity and selectivity of DesAb-O for Aβ_42_ oligomers, at least equal to that of the A11 and 19.3 Abs, raised against prefibrillar oligomers and ADDLs, respectively [16, 18], with only a minor cross-reaction with the monomeric protein, nontoxic oligomers and fibrillar conformers. We also evaluated the ability of DesAb-O to selectively detect Aβ_42_ oligomers in cultured cells, demonstrating a great performance also in a more physiological condition.

We then revealed that the pre-incubation of DesAb-O with Aβ_42_ oligomers strongly reduces their interaction with neuronal membranes in a dose-dependent manner, and this protective effect appears more evident for DesAb-O than the A11 Ab, at least at low Aβ_42_:Abs molar ratios. This suggests that DesAb-O can detect the key epitopes normally exposed on the surface of toxic oligomers and responsible for their interaction with the membrane more effectively than the A11 Ab, presumably because of its smaller size, that enables a more precise targeting of critical epitopes. Our results are consistent with those obtained with conformation-sensitive Abs, such as ACU-954 and A-887755, specifically raised against Aβ oligomers, namely ADDLs and globulomers, respectively, that were found to prevent their binding to neurons [21, 69], rescuing the impaired synaptic transmission [21]. The capture of Aβ_42_ oligomers by DesAb-O was also found to prevent their induced mitochondrial dysfunction, in agreement with a large body of evidence supporting the protective effects of Abs, such as A11, OC, AUC-954, A-887755 and PMN310, against neuronal dysfunction [18, 20, 21, 45, 69, 70]. Notably, DesAb-O showed no inherent toxicity when added alone to the cell culture medium, thus making it an excellent tool for future tentative therapeutic applications.

Other sdAbs have been reported to target pathologically relevant Aβ_42_ oligomers. For example, the V31-1 was able to recognise intraneuronal oligomers in human brain slices and to inhibit fibril formation preventing their induced neurotoxicity [64], suggesting a great potential for the detection and diagnosis of Aβ oligomers in AD patients [71]. In addition, two sdAbs, namely PrioAD12 against Aβ_40_ and PrioAD13 against Aβ_42_ [66] can detect Aβ oligomers simultaneously in the blood and retina of APP/PS1 mice before their appearance in the brain, with respect to age-matched wild-type mice controls [72].

When we moved our approach to CSFs, we revealed a remarkable ability of DesAb-O to selectively identify Aβ_42_ oligomers also in a biological fluid from AD patients compared to age-matched control subjects. Previous studies detected Aβ_42_ oligomers in AD CSFs by using two-site ELISA assay and the couple 19.3/82E1 Abs [16], or homotypic ELISA using 82E1 Ab [73] or BAN50 Ab [74]. Even if these reported assays used different oligomer standards, a trend has emerged suggesting that a sub-pg/ml sensitivity is required to detect CSF oligomers [16, 73, 75]. Indeed, in our assay we have been able to measure an average oligomer concentration of c.a. 4.5 pg/ml, in good agreement with previous findings [16, 73, 76]. It is important to clarify that two studies reported a lack of significant change in total aggregates between AD and controls, using both a sdAb, named Nb3, and a mAb, named Bapineuzimab [76, 77]. This suggests that the total amount of the aggregates is not a critical factor in AD CSFs, but it is rather the nature of these aggregates and their effects to be important.

Further analyses were conducted in this work on neuroblastoma cells to test the possible therapeutic potential of DesAb-O against the neurotoxic Aβ_42_ oligomers present in the CSFs of AD patients. We first demonstrated that the CSFs of AD patients caused significant Ca^2+^ influx, membrane permeabilisation and mitochondrial dysfunction, in agreement with previous findings [19, 78–83], and suggesting that the AD CSF contains neurotoxic species. Notably, DesAb-O was found to prevent neuronal dysfunction caused by Aβ oligomers present in the CSFs of AD patients, in agreement with other studies using Nb3 and other Abs against Aβ [79, 80], or extracellular chaperones [82]. It is well known that the shielding of toxic hydrophobic regions exposed on the oligomer surface by either polyclonal or monoclonal Abs, extracellular chaperones and even other proteins present in biological fluids such as transthyretin, appears to be an effective method to suppress the toxicity of misfolded Aβ_42_ oligomers [16, 18, 84, 85], but the high sensitivity and selectivity of DesAb-O and possibly other sdAbs relative to other Abs and chaperones could offer a remarkable potential of these biotechnological tools against AD particularly in its early stages.

While additional studies are required in larger cohorts of AD patients and controls, our data support the idea that sdAbs raised against Aβ_42_ oligomers could represent a new biotech tool for diagnosing AD in the very early stages of pathology. Moreover, our evidence offers the possibility to explore new therapeutic strategies based on sdAbs for AD and other protein misfolding diseases.

## Conclusions

In this work, we demonstrate a great ability of a single-domain antibody (sdAb), named DesAb-O, to selectively identify and counteract Aβ_42_ oligomers in a biologically and relevant heterogenous context, such as the cerebrospinal fluid from AD patients with respect to healthy subjects, taking advantage of an array of different techniques such as dot-blot, ELISA immunoassay, super-resolution STED microscopy and in-cell analyses. Taken together, our data indicate a promising method for the improvement of an early diagnosis of AD and for the generation of novel therapeutic approaches based on sdAbs for the treatment of AD and other devastating neurodegenerative conditions.

## Supplementary Information

**Additional file 1:** **Figure S1.** 6E10 Ab detects all Aβ_42 _species in a dose-dependent manner. Indirect ELISA measurements taken at increasing concentration of Aβ_42 _species using the 6E10 Ab. Data were normalised for the corresponding average value at concentration 0 μM. Experimental errors are S.D. (*n*=3). Samples were analysed by Student *t* test relative to 0 μM (*P<0.05, ** *P*<0.01 and ***P<0.001). **Figure S2. **DesAb-O detects Aβ_42_ oligomers bound to the neuronal membrane and internalized into the cytosol.Representative STED microscopy images showing the basal, median, and apical sections of SH-SY5Y cells treated for 1 h with the indicated Aβ_42_ species at 3.0 μM (monomer equivalents) for 1 h. Red and green fluorescence indicates respectively the cell membranes and the Aβ_42_ species, detected with WGA and DesAb-O Ab. **Figure S3.** DesAb18–24 detects Aβ_42_ fibrils and shows a non-specific signal in the CSF samples (A) Indirect sandwich ELISA assay. 0.25 mg/ml of CSF samples from AD patients and control subjects were adsorbed and quantified using DesAb18–24 at 0.5 µM. Standard curve was obtained with decreasing concentration of Aβ_42_ species formed *in vitro*. Data were normalized for the corresponding average value at concentration 0 pg/ml). Experimental errors are S.E.M. (*n*=4). Samples were analysed by Student *t* test relative to 0 pg/ml (* *P*<0.05, **P<0.01, *** *P*<0.001) or to F1 (§ *P*<0.05,§§ *P*<0.01, §§§*P*<0.001) or to control CSF (°° *P*<0.01). (B) Representative STED images showing Aβ_42_ species (M, A+ oligomers, F1, and a mixture containing both A+ and F1 at 1:1 molar ratio) and CSFs collected from AD patients and controls (*n*=4) spotted in a glass coverslip at 25 µM and 0.5 mg/ml, respectively. The green fluorescent signals arise from the staining with DesAb18–24.

## Data Availability

The authors confirm that all data needed to evaluate the conclusions of this study are available within the article.

## Abbreviations

A.T.C.C.: American Type Culture Collection
Ab: Antibody
AD: Alzheimer’s disease
ADDLs: Amyloid β-derived diffusible ligands
AFM: Atomic force microscopy
APP: Amyloid precursor protein
Aβ_42_: Amyloid beta 42
BSA: Bovine serum albumin
Calcein-AM: Calcein-acetoxymethyl
CSF: Cerebrospinal fluid
CSFs: Cerebrospinal fluid samples
DMEM: Dulbecco’s modified Eagle’s medium
DMSO: Dimethyl sulfoxide
EDTA: Ethylenediamine tetraacetic acid
ELISA: Enzyme-linked immunosorbent assay
FBS: Fetal bovine serum
FDA: Food and Drug Administration
FITC: Fluorescein isothiocyanate
Fluo-4 AM: Fluo-4 acetoxymethyl
H_2_SO_4_: Sulfuric acid
HCl: Hydrogen chloride
HEPES: 4-(2-Hydroxyethyl) piperazine-1-ethanesulfonic acid
HFIP: Hexafluoro-2-isopropanol
HRP: Horseradish peroxidase
mAb: Monoclonal antibody
MCI: Mild cognitive impairment
MTT: 3-(4,5-Dimethylthiazol-2-yl)-2,5-diphenyltetrazolium bromide
NaCl: Sodium chloride
NaOH: Sodium hydroxide
PBS: Phosphate buffered saline
RT: Room temperature
S.D.: Standard deviation
S.E.M: Standard error of the mean
sdAb: Single-domain antibody
STED: Stimulated emission depletion
TBS: Tris buffered saline
Tris: Tris(hydroxymethyl)aminomethane
VHH: Variable heavy domain of heavy chain
WGA: Wheat germ agglutinin
αSyn: α-Synuclein

## References

[1] Mucke L. Neuroscience: Alzheimer’s disease. Nature. 2009;461(7266):895–7. 19829367 10.1038/461895a

[2] Rajmohan R, Reddy PH. Aβ and phosphorylated tau accumulations cause abnormalities at synapses of Alzheimer’s disease neurons. J Alzheimers Dis. 2017;57(4):975–99. 27567878 10.3233/JAD-160612 PMC5793225

[3] Patterson C. World Alzheimer Report. The state of the art of dementia research: new frontiers. Alzheimer’s Disease International. 2018;2018:1–48.

[4] Kayed R, Sokolov Y, Edmonds B, McIntire TM, Milton SC, Hall JE, Glabe CG. Permeabilization of lipid bilayers is a common conformation-dependent activity of soluble amyloid oligomers in protein misfolding diseases. J Biol Chem. 2004;279(45):46363–6. 15385542 10.1074/jbc.C400260200

[5] Benilova I, Karran E, De Strooper B. The toxic Aβ oligomer and Alzheimer’s disease: an emperor in need of clothes. Nat Neurosci. 2012;15(3):349–57. 22286176 10.1038/nn.3028

[6] Evangelisti E, Cascella R, Becatti M, Marrazza G, Dobson CM, Chiti F, Stefani M, Cecchi C. Binding affinity of amyloid oligomers to cellular membranes is a generic indicator of cellular dysfunction in protein misfolding diseases. Sci Rep. 2016;6:32721. 27619987 10.1038/srep32721 PMC5020652

[7] Bigi A, Cascella R, Fani G, Bernacchioni C, Cencetti F, Bruni P, Chiti F, Donati C, Cecchi C. Sphingosine 1-phosphate attenuates neuronal dysfunction induced by Aβ oligomers through endocytic internalization of NMDA receptors. FEBS J. 2023;290(1):112–33. 35851748 10.1111/febs.16579 PMC10087929

[8] Cline EN, Bicca MA, Viola KL, Klein WL. The Aβ oligomer hypothesis: beginning of the third decade. J Alzheimers Dis. 2018;64(s1):S567–610. 29843241 10.3233/JAD-179941 PMC6004937

[9] Selkoe DJ. Alzheimer disease and aducanumab: adjusting our approach. Nat Rev Neurol. 2019;15(7):365–6. 31138932 10.1038/s41582-019-0205-1

[10] Westerman MA, Cooper-Blacketer D, Mariash A, Kotilinek L, Kawarabayashi T, Younkin LH, Carlson GA, Younkin SG, Ashe KH. The relationship between Aβ and memory in the Tg2576 mouse model of Alzheimer’s disease. J Neurosci. 2002;22(5):1858–67. 11880515 10.1523/JNEUROSCI.22-05-01858.2002 PMC6758862

[11] Selkoe DJ. Cell biology of protein misfolding: the examples of Alzheimer’s and Parkinson’s diseases. Nat Cell Biol. 2004;6(11):1054–61. 15516999 10.1038/ncb1104-1054

[12] Hardy JA, Higgins GA. Alzheimer’s disease: the amyloid cascade hypothesis. Science. 1992;256(5054):184–5. 1566067 10.1126/science.1566067

[13] Limbocker R, Cremades N, Cascella R, Tessier PM, Vendruscolo M, Chiti F. Characterization of pairs of toxic and nontoxic misfolded protein oligomers elucidates the structural determinants of oligomer toxicity in protein misfolding diseases. Acc Chem Res. 2023;56(12):1395–405. 37071750 10.1021/acs.accounts.3c00045 PMC10286310

[14] Haass C, Selkoe DJ. Soluble protein oligomers in neurodegeneration: lessons from the Alzheimer’s amyloid beta-peptide. Nat Rev Mol Cell Biol. 2007;8(2):101–12. 17245412 10.1038/nrm2101

[15] Hefti F, Goure WF, Jerecic J, Iverson KS, Walicke PA, Krafft GA. The case for soluble Aβ oligomers as a drug target in Alzheimer’s disease. Trends Pharmacol Sci. 2013;34(5):261–6. 23582316 10.1016/j.tips.2013.03.002

[16] Savage MJ, Kalinina J, Wolfe A, Tugusheva K, Korn R, Cash-Mason T, Maxwell JW, Hatcher NG, Haugabook SJ, Wu G, Howell BJ, Renger JJ, Shughrue PJ, McCampbell A. A sensitive Aβ oligomer assay discriminates Alzheimer’s and aged control cerebrospinal fluid. J Neurosci. 2014;34(8):2884–97. 24553930 10.1523/JNEUROSCI.1675-13.2014 PMC6608513

[17] De S, Whiten DR, Ruggeri FS, Hughes C, Rodrigues M, Sideris DI, Taylor CG, Aprile FA, Muyldermans S, Knowles TPJ, Vendruscolo M, Bryant C, Blennow K, Skoog I, Kern S, Zetterberg H, Klenerman D. Soluble aggregates present in cerebrospinal fluid change in size and mechanism of toxicity during Alzheimer’s disease progression. Acta Neuropathol Commun. 2019;7(1):120. 31349874 10.1186/s40478-019-0777-4 PMC6659275

[18] Kayed R, Head E, Thompson JL, McIntire TM, Milton SC, Cotman CW, Glabe CG. Common structure of soluble amyloid oligomers implies common mechanism of pathogenesis. Science. 2003;300(5618):486–9. 12702875 10.1126/science.1079469

[19] Gong Y, Chang L, Viola KL, Lacor PN, Lambert MP, Finch CE, Krafft GA, Klein WL. Alzheimer’s disease-affected brain: presence of oligomeric Aβ ligands (ADDLs) suggests a molecular basis for reversible memory loss. Proc Natl Acad Sci U S A. 2003;100(18):10417–22. 12925731 10.1073/pnas.1834302100 PMC193576

[20] Kayed R, Head E, Sarsoza F, Saing T, Cotman CW, Necula M, Margol L, Wu J, Breydo L, Thompson JL, Rasool S, Gurlo T, Butler P, Glabe CG. Fibril specific, conformation dependent antibodies recognize a generic epitope common to amyloid fibrils and fibrillar oligomers that is absent in prefibrillar oligomers. Mol Neurodegener. 2007;2:18. 17897471 10.1186/1750-1326-2-18 PMC2100048

[21] Hillen H, Barghorn S, Striebinger A, Labkovsky B, Müller R, Nimmrich V, Nolte MW, Perez-Cruz C, van der Auwera I, van Leuven F, van Gaalen M, Bespalov AY, Schoemaker H, Sullivan JP, Ebert U. Generation and therapeutic efficacy of highly oligomer-specific beta-amyloid antibodies. J Neurosci. 2010;30(31):10369–79. 20685980 10.1523/JNEUROSCI.5721-09.2010 PMC6634649

[22] Hof PR, Giannakopoulos P, Bouras C. The neuropathological changes associated with normal brain aging. Histol Histopathol. 1996;11:1075–88. 8930649

[23] Perl DP. Neuropathology of Alzheimer’s disease. Mt Sinai J Med. 2010;77:32–42. 20101720 10.1002/msj.20157 PMC2918894

[24] Bateman RJ, Xiong C, Benzinger TL, Fagan AM, Goate A, Fox NC, Marcus DS, Cairns NJ, Xie X, Blazey TM, Holtzman DM, Santacruz A, Buckles V, Oliver A, Moulder K, Aisen PS, Ghetti B, Klunk WE, McDade E, Martins RN, et al. Clinical and biomarker changes in dominantly inherited Alzheimer’s disease. N Engl J Med. 2012;367:795–804. 22784036 10.1056/NEJMoa1202753 PMC3474597

[25] Vaz M, Silvestre S. Alzheimer’s disease: Recent treatment strategies. Eur J Pharmacol. 2020;887: 173554. 32941929 10.1016/j.ejphar.2020.173554

[26] Karran E, De Strooper B. The amyloid hypothesis in Alzheimer disease: new insights from new therapeutics. Nat Rev Drug Discov. 2022;21(4):306–18. 35177833 10.1038/s41573-022-00391-w

[27] Sevigny J, Chiao P, Bussière T, Weinreb PH, Williams L, Maier M, Dunstan R, Salloway S, Chen T, Ling Y, et al. The antibody aducanumab reduces Aβ plaques in Alzheimer’s disease. Nature. 2016;537(7618):50–6. 27582220 10.1038/nature19323

[28] van Dyck CH, Swanson CJ, Aisen P, Bateman RJ, Chen C, Gee M, Kanekiyo M, Li D, Reyderman L, Cohen S, Froelich L, Katayama S, Sabbagh M, Vellas B, Watson D, Dhadda S, Irizarry M, Kramer LD, Iwatsubo T. Lecanemab in Early Alzheimer’s Disease. N Engl J Med. 2023;388(1):9–21. 36449413 10.1056/NEJMoa2212948

[29] Budd Haeberlein S, Aisen PS, Barkhof F, Chalkias S, Chen T, Cohen S, Dent G, Hansson O, Harrison K, von Hehn C, Iwatsubo T, et al. Two randomized phase 3 studies of aducanumab in early Alzheimer’s disease. J Prev Alzheimers Dis. 2022;9(2):197–210. 35542991 10.14283/jpad.2022.30

[30] Leisher S, Bohorquez A, Gay M, Garcia V, Jones R, Baldaranov D, Rafii MS. Amyloid-lowering monoclonal antibodies for the treatment of early Alzheimer’s disease. CNS Drugs. 2023;37(8):671–7. 37470978 10.1007/s40263-023-01021-8 PMC10439019

[31] Ackaert C, Smiejkowska N, Xavier C, Sterckx YGJ, Denies S, Stijlemans B, Elkrim Y, Devoogdt N, Caveliers V, Lahoutte T, Muyldermans S, Breckpot K, Keyaerts M. Immunogenicity risk profile of nanobodies. Front Immunol. 2021;12: 632687. 33767701 10.3389/fimmu.2021.632687 PMC7985456

[32] Olzscha H, Schermann SM, Woerner AC, Pinkert S, Hecht MH, Tartaglia GG, Vendruscolo M, Hayer-Hartl M, Hartl FU, Vabulas RM. Amyloid-like aggregates sequester numerous metastable proteins with essential cellular functions. Cell. 2011;144(1):67–78. 21215370 10.1016/j.cell.2010.11.050

[33] Muyldermans S. Nanobodies: natural single-domain antibodies. Annu Rev Biochem. 2013;82:775–97. 23495938 10.1146/annurev-biochem-063011-092449

[34] Aprile FA, Sormanni P, Perni M, Arosio P, Linse S, Knowles TPJ, Dobson CM, Vendruscolo M. Selective targeting of primary and secondary nucleation pathways in Aβ_42_ aggregation using a rational antibody scanning method. Sci Adv. 2017;3(6): e1700488. 28691099 10.1126/sciadv.1700488 PMC5479649

[35] Aprile FA, Sormanni P, Podpolny M, Chhangur S, Needham LM, Ruggeri FS, Perni M, Limbocker R, Heller GT, Sneideris T, et al. Rational design of a conformation-specific antibody for the quantification of Aβ oligomers. Proc Natl Acad Sci USA. 2020;117:13509–18. 32493749 10.1073/pnas.1919464117 PMC7306997

[36] Sormanni P, Aprile FA, Vendruscolo M. Rational design of antibodies targeting specific epitopes within intrinsically disordered proteins. Proc Natl Acad Sci U S A. 2015;112(32):9902–7. 26216991 10.1073/pnas.1422401112 PMC4538631

[37] Sormanni P, Aprile FA, Vendruscolo M. Third generation antibody discovery methods: in silico rational design. Chem Soc Rev. 2018;47(24):9137–57. 30298157 10.1039/c8cs00523k

[38] Lambert MP, Barlow AK, Chromy BA, Edwards C, Freed R, Liosatos M, Morgan TE, Rozovsky I, Trommer B, Viola KL, Wals P, Zhang C, Finch CE, Krafft GA, Klein WL. Diffusible, nonfibrillar ligands derived from Aβ are potent central nervous system neurotoxins. Proc Natl Acad Sci U S A. 1998;95(11):6448–53. 9600986 10.1073/pnas.95.11.6448 PMC27787

[39] Dahlgren KN, Manelli AM, Stine WB Jr, Baker LK, Krafft GA, LaDu MJ. Oligomeric and fibrillar species of Aβ peptides differentially affect neuronal viability. J Biol Chem. 2002;277(35):32046–53. 12058030 10.1074/jbc.M201750200

[40] Ladiwala AR, Litt J, Kane RS, Aucoin DS, Smith SO, Ranjan S, Davis J, Van Nostrand WE, Tessier PM. Conformational differences between two Aβ oligomers of similar size and dissimilar toxicity. J Biol Chem. 2012;287(29):24765–73. 22547072 10.1074/jbc.M111.329763 PMC3397903

[41] Bradford MM. A rapid and sensitive method for the quantitation of microgram quantities of protein utilizing the principle of protein-dye binding. Anal Biochem. 1976;72:248–54. 942051 10.1016/0003-2697(76)90527-3

[42] Capitini C, Bigi A, Parenti N, Emanuele M, Bianchi N, Cascella R, Cecchi C, Maggi L, Annunziato F, Pavone FS, Calamai M. APP and Bace1: Differential effect of cholesterol enrichment on processing and plasma membrane mobility. iScience. 2023;26(5):106611. 10.1016/j.isci.2023.106611 PMC10148118 37128606

[43] Fani G, La Torre CE, Cascella R, Cecchi C, Vendruscolo M, Chiti F. Misfolded protein oligomers induce an increase of intracellular Ca^2+^ causing an escalation of reactive oxidative species. Cell Mol Life Sci. 2022;79(9):500. 36030306 10.1007/s00018-022-04513-w PMC9420098

[44] Cascella R, Chen SW, Bigi A, Camino JD, Xu CK, Dobson CM, Chiti F, Cremades N, Cecchi C. The release of toxic oligomers from α-synuclein fibrils induces dysfunction in neuronal cells. Nat Commun. 202;12(1):1814. 10.1038/s41467-021-21937-3 PMC7985515 33753734

[45] Bigi A, Loffredo G, Cascella R, Cecchi C. Targeting pathological amyloid aggregates with conformation-sensitive antibodies. Curr Alzheimer Res. 2020;17(8):722–34. 33167834 10.2174/1567205017666201109093848

[46] Mosmann T. Rapid colorimetric assay for cellular growth and survival: application to proliferation and cytotoxicity assays. J Immunol Methods. 1983;65(1–2):55–63. 6606682 10.1016/0022-1759(83)90303-4

[47] Evangelisti E, Zampagni M, Cascella R, Becatti M, Fiorillo C, Caselli A, Bagnoli S, Nacmias B, Cecchi C. Plasma membrane injury depends on bilayer lipid composition in Alzheimer’s disease. J Alzheimers Dis. 2014;41(1):289–300. 24614900 10.3233/JAD-131406

[48] Cascella R, Evangelisti E, Bigi A, Becatti M, Fiorillo C, Stefani M, Chiti F, Cecchi C. Soluble oligomers require a ganglioside to trigger neuronal calcium overload. J Alzheimers Dis. 2017;60(3):923–38. 28922156 10.3233/JAD-170340

[49] Banchelli M, Cascella R, D’Andrea C, Cabaj L, Osticioli I, Ciofini D, Li MS, Skupień K, de Angelis M, Siano S, Cecchi C, Pini R, La Penna G, Chiti F, Matteini P. Nanoscopic insights into the surface conformation of neurotoxic Aβ oligomers. RSC Adv. 2020;10(37):21907–13. 35516647 10.1039/d0ra03799k PMC9054531

[50] Kim KS, Wen GY, Bancher C, Chen CMJ, Sapienza V, Hong H, Wisniewski HM. Detection and quantification of amyloid β-peptide with two monoclonal antibodies. Neurosci Res Comm. 1990;7:113–22.

[51] Baghallab I, Reyes-Ruiz JM, Abulnaja K, Huwait E, Glabe C. Epitomic characterization of the specificity of the anti-amyloid aβ monoclonal antibodies 6e10 and 4g8. J Alzheimers Dis. 2018;66(3):1235–44. 30412489 10.3233/JAD-180582 PMC6294585

[52] Schengrund CL. Lipid rafts: keys to neurodegeneration. Brain Res Bull. 2010;82(1–2):7–17. 20206240 10.1016/j.brainresbull.2010.02.013

[53] Evangelisti E, Wright D, Zampagni M, Cascella R, Fiorillo C, Bagnoli S, Relini A, Nichino D, Scartabelli T, Nacmias B, Sorbi S, Cecchi C. Lipid rafts mediate amyloid-induced calcium dyshomeostasis and oxidative stress in Alzheimer’s disease. Curr Alzheimer Res. 2013;10(2):143–53. 22950913 10.2174/1567205011310020004

[54] Demuro A, Mina E, Kayed R, Milton SC, Parker I, Glabe CG. Calcium dysregulation and membrane disruption as a ubiquitous neurotoxic mechanism of soluble amyloid oligomers. J Biol Chem. 2005;280:17294–300. 15722360 10.1074/jbc.M500997200

[55] Arbel-Ornath M, Hudry E, Boivin JR, Hashimoto T, Takeda S, Kuchibhotla KV, Hou S, Lattarulo CR, Belcher AM, Shakerdge N, et al. Soluble oligomeric amyloid-β induces calcium dyshomeostasis that precedes synapse loss in the living mouse brain. Mol Neurodegener. 2017;12(1):27. 28327181 10.1186/s13024-017-0169-9 PMC5361864

[56] Cascella R, Cecchi C. Calcium dyshomeostasis in Alzheimer’s disease pathogenesis. Int J Mol Sci. 2021;22:4914. 34066371 10.3390/ijms22094914 PMC8124842

[57] Bigi A, Lombardo E, Cascella R, Cecchi C. The toxicity of protein aggregates: new insights into the mechanisms. Int J Mol Sci. 2023;24(9):7974. 37175681 10.3390/ijms24097974 PMC10178715

[58] Li S, Jin M, Liu L, Dang Y, Ostaszewski BL, Selkoe DJ. Decoding the synaptic dysfunction of bioactive human AD brain soluble Aβ to inspire novel therapeutic avenues for Alzheimer’s disease. Acta Neuropathol Commun. 2018;6(1):121. 30409172 10.1186/s40478-018-0626-x PMC6225562

[59] Jekel K, Damian M, Wattmo C, Hausner L, Bullock R, Connelly PJ, Dubois B, Eriksdotter M, Ewers M, Graessel E, et al. Mild cognitive impairment and deficits in instrumental activities of daily living: a systematic review. Alzheimers Res Ther. 2015;7(1):17. 25815063 10.1186/s13195-015-0099-0 PMC4374414

[60] Grant MKO, Handoko M, Rozga M, Brinkmalm G, Portelius E, Blennow K, Ashe KH, Zahs KR, Liu P. Human cerebrospinal fluid 6E10-immunoreactive protein species contain amyloid precursor protein fragments. PLoS ONE. 2019;14(2): e0212815. 30817799 10.1371/journal.pone.0212815 PMC6394962

[61] Sideris DI, Danial JSH, Emin D, Ruggeri FS, Xia Z, Zhang YP, Lobanova E, Dakin H, De S, Miller A, Sang JC, et al. Soluble amyloid beta-containing aggregates are present throughout the brain at early stages of Alzheimer's disease. Brain Commun. 2021;3(3):fcab147. 10.1093/braincomms/fcab147 PMC8361392 34396107

[62] Zheng F, Pang Y, Li L, Pang Y, Zhang J, Wang X, Raes G. Applications of nanobodies in brain diseases. Front Immunol. 2022;13: 978513. 36426363 10.3389/fimmu.2022.978513 PMC9679430

[63] Zameer A, Kasturirangan S, Emadi S, Nimmagadda SV, Sierks MR. Anti-oligomeric Aβ single-chain variable domain antibody blocks Abeta-induced toxicity against human neuroblastoma cells. J Mol Biol. 2008;384(4):917–28. 18929576 10.1016/j.jmb.2008.09.068

[64] Lafaye P, Achour I, England P, Duyckaerts C, Rougeon F. Single-domain antibodies recognize selectively small oligomeric forms of Aβ, prevent Aβ -induced neurotoxicity and inhibit fibril formation. Mol Immunol. 2009;46(4):695–704. 18930548 10.1016/j.molimm.2008.09.008

[65] Kasturirangan S, Li L, Emadi S, Boddapati S, Schulz P, Sierks MR. Nanobody specific for oligomeric Aβ stabilizes nontoxic form. Neurobiol Aging. 2012;33(7):1320–8. 21067847 10.1016/j.neurobiolaging.2010.09.020

[66] David MA, Jones DR, Tayebi M. Potential candidate camelid antibodies for the treatment of protein-misfolding diseases. J Neuroimmunol. 2014;272(1–2):76–85. 24864011 10.1016/j.jneuroim.2014.05.001

[67] Bélanger K, Iqbal U, Tanha J, MacKenzie R, Moreno M, Stanimirovic D. Single-domain antibodies as therapeutic and imaging agents for the treatment of cns diseases. Antibodies (Basel). 2019;8(2):27. 31544833 10.3390/antib8020027 PMC6640712

[68] Li T, Vandesquille M, Koukouli F, Dudeffant C, Youssef I, Lenormand P, Ganneau C, Maskos U, Czech C, Grueninger F, Duyckaerts C, Dhenain M, Bay S, Delatour B, Lafaye P. Camelid single-domain antibodies: A versatile tool for in vivo imaging of extracellular and intracellular brain targets. J Control Release. 2016;243:1–10. 27671875 10.1016/j.jconrel.2016.09.019

[69] Shughrue PJ, Acton PJ, Breese RS, Zhao WQ, Chen-Dodson E, Hepler RW, Wolfe AL, Matthews M, Heidecker GJ, Joyce JG, Villarreal SA, Kinney GG. Anti-ADDL antibodies differentially block oligomer binding to hippocampal neurons. Neurobiol Aging. 2010;31(2):189–202. 18486276 10.1016/j.neurobiolaging.2008.04.003

[70] Gibbs E, Silverman JM, Zhao B, Peng X, Wang J, Wellington CL, Mackenzie IR, Plotkin SS, Kaplan JM, Cashman NR. A rationally designed humanized antibody selective for Aβ oligomers in Alzheimer’s disease. Sci Rep. 2019;9(1):9870. 31285517 10.1038/s41598-019-46306-5 PMC6614461

[71] Pain C, Dumont J, Dumoulin M. Camelid single-domain antibody fragments: Uses and prospects to investigate protein misfolding and aggregation, and to treat diseases associated with these phenomena. Biochimie. 2015;111:82–106. 25656912 10.1016/j.biochi.2015.01.012

[72] Habiba U, Descallar J, Kreilaus F, Adhikari UK, Kumar S, Morley JW, Bui BV, Koronyo-Hamaoui M, Tayebi M. Detection of retinal and blood Aβ oligomers with nanobodies. Alzheimers Dement (Amst). 2021;13(1): e12193. 33977118 10.1002/dad2.12193 PMC8101010

[73] Hölttä M, Hansson O, Andreasson U, Hertze J, Minthon L, Nägga K, Andreasen N, Zetterberg H, Blennow K. Evaluating Aβ oligomers in cerebrospinal fluid as a biomarker for Alzheimer’s disease. PLoS ONE. 2013;8(6): e66381. 23799095 10.1371/journal.pone.0066381 PMC3682966

[74] Herskovits AZ, Locascio JJ, Peskind ER, Li G, Hyman BT. A Luminex assay detects Aβ oligomers in Alzheimer’s disease cerebrospinal fluid. PLoS ONE. 2013;8(7): e67898. 23844122 10.1371/journal.pone.0067898 PMC3699502

[75] Georganopoulou DG, Chang L, Nam JM, Thaxton CS, Mufson EJ, Klein WL, Mirkin CA. Nanoparticle-based detection in cerebral spinal fluid of a soluble pathogenic biomarker for Alzheimer’s disease. Proc Natl Acad Sci U S A. 2005;102(7):2273–6. 15695586 10.1073/pnas.0409336102 PMC548981

[76] Yang T, O’Malley TT, Kanmert D, Jerecic J, Zieske LR, Zetterberg H, Hyman BT, Walsh DM, Selkoe DJ. A highly sensitive novel immunoassay specifically detects low levels of soluble Aβ oligomers in human cerebrospinal fluid. Alzheimers Res Ther. 2015;7(1):14. 25802556 10.1186/s13195-015-0100-y PMC4369838

[77] Drews A, De S, Flagmeier P, Wirthensohn DC, Chen WH, Whiten DR, Rodrigues M, Vincke C, Muyldermans S, Paterson RW, Slattery CF, Fox NC, Schott JM, Zetterberg H, Dobson CM, Gandhi S, Klenerman D. Inhibiting the Ca^2+^ influx induced by human CSF Cell Rep. 2017;21(11):3310–3316. 10.1016/j.celrep.2017.11.057 PMC5745229 29241555

[78] Näslund J, Haroutunian V, Mohs R, Davis KL, Davies P, Greengard P, Buxbaum JD. Correlation between elevated levels of Aβ -peptide in the brain and cognitive decline. JAMA. 2000;283(12):1571–7. 10735393 10.1001/jama.283.12.1571

[79] Walsh DM, Klyubin I, Fadeeva JV, Cullen WK, Anwyl R, Wolfe MS, Rowan MJ, Selkoe DJ. Naturally secreted oligomers of Aβ protein potently inhibit hippocampal long-term potentiation in vivo. Nature. 2002;416(6880):535–9. 11932745 10.1038/416535a

[80] Klyubin I, Betts V, Welzel AT, Blennow K, Zetterberg H, Wallin A, Lemere CA, Cullen WK, Peng Y, Wisniewski T, et al. Aβ protein dimer-containing human CSF disrupts synaptic plasticity: prevention by systemic passive immunization. J Neurosci. 2008;28:4231–7. 18417702 10.1523/JNEUROSCI.5161-07.2008 PMC2685151

[81] Demuro A, Parker I, Stutzmann GE. Calcium signaling and amyloid toxicity in Alzheimer disease. J Biol Chem. 2010;285(17):12463–8. 20212036 10.1074/jbc.R109.080895 PMC2857063

[82] Yerbury JJ, Wilson MR. Extracellular chaperones modulate the effects of Alzheimer’s patient cerebrospinal fluid on Aβ_42_ toxicity and uptake. Cell Stress Chaperones. 2010;15(1):115–21. 19472074 10.1007/s12192-009-0122-0 PMC2866977

[83] Lee SJ, Nam E, Lee HJ, Savelieff MG, Lim MH. Towards an understanding of Aβ oligomers: characterization, toxicity mechanisms, and inhibitors. Chem Soc Rev. 2017;46(2):310–23. 27878186 10.1039/c6cs00731g

[84] Cascella R, Conti S, Tatini F, Evangelisti E, Scartabelli T, Casamenti F, Wilson MR, Chiti F, Cecchi C. Extracellular chaperones prevent Aβ_42_-induced toxicity in rat brains. Biochim Biophys Acta. 2013;1832(8):1217–26. 23602994 10.1016/j.bbadis.2013.04.012

[85] Cascella R, Conti S, Mannini B, Li X, Buxbaum JN, Tiribilli B, Chiti F, Cecchi C. Transthyretin suppresses the toxicity of oligomers formed by misfolded proteins in vitro. Biochim Biophys Acta. 2013;1832(12):2302–14. 24075940 10.1016/j.bbadis.2013.09.011

