# Supplementary material for: A single-domain antibody detects and neutralises toxic Aβ42 oligomers in the Alzheimer’s disease CSF
Source: Alzheimers Res Ther. 2024 Jan 18;16:13. doi: 10.1186/s13195-023-01361-z (PMC10795411; doi:10.1186/s13195-023-01361-z)
Supplement: Supplementary file 1 — Additional file 1: Figure S1. 6E10 Ab detects all Aβ42 species in a dose-dependent manner. Indirect ELISA measurements taken at increasing concentration of Aβ42 species using the 6E10 Ab. Data were normalised for the corresponding average value at concentration 0 μM. Experimental errors are S.D. (n=3). Samples were analysed by Student t test relative to 0 μM (*P<0.05, ** P<0.01 and ***P<0.001). Figure S2. DesAb-O detects Aβ42 oligomers bound to the neuronal membrane and internalized into the cytosol.Representative STED microscopy images showing the basal, median, and apical sections of SH-SY5Y cells treated for 1 h with the indicated Aβ42 species at 3.0 μM (monomer equivalents) for 1 h. Red and green fluorescence indicates respectively the cell membranes and the Aβ42 species, detected with WGA and DesAb-O Ab. Figure S3. DesAb18–24 detects Aβ42 fibrils and shows a non-specific signal in the CSF samples (A) Indirect sandwich ELISA assay. 0.25 mg/ml of CSF samples from AD patients and control subjects were adsorbed and quantified using DesAb18–24 at 0.5 µM. Standard curve was obtained with decreasing concentration of Aβ42 species formed in vitro. Data were normalized for the corresponding average value at concentration 0 pg/ml). Experimental errors are S.E.M. (n=4). Samples were analysed by Student t test relative to 0 pg/ml (* P<0.05, **P<0.01, *** P<0.001) or to F1 (§ P<0.05,§§ P<0.01, §§§P<0.001) or to control CSF (°° P<0.01). (B) Representative STED images showing Aβ42 species (M, A+ oligomers, F1, and a mixture containing both A+ and F1 at 1:1 molar ratio) and CSFs collected from AD patients and controls (n=4) spotted in a glass coverslip at 25 µM and 0.5 mg/ml, respectively. The green fluorescent signals arise from the staining with DesAb18–24. [file 13195_2023_1361_MOESM1_ESM.docx]

**Supplementary figures and legends**


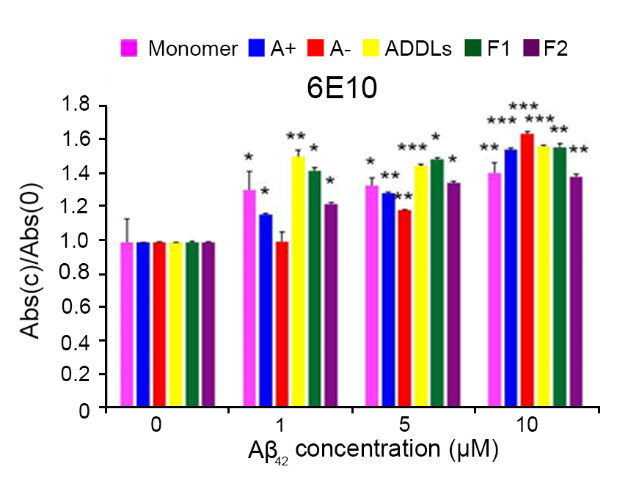


**Figure S1.** **6E10 Ab detects all Aβ_42_species in a dose-dependent manner.** Indirect ELISA measurements taken at increasing concentration of Aβ_42_species using the 6E10 Ab. Data were normalised for the corresponding average value at concentration 0 μM. Experimental errors are S.D. (n=3). Samples were analysed by Student *t* test relative to 0 μM (*P<0.05, ** P<0.01 and ***P<0.001).


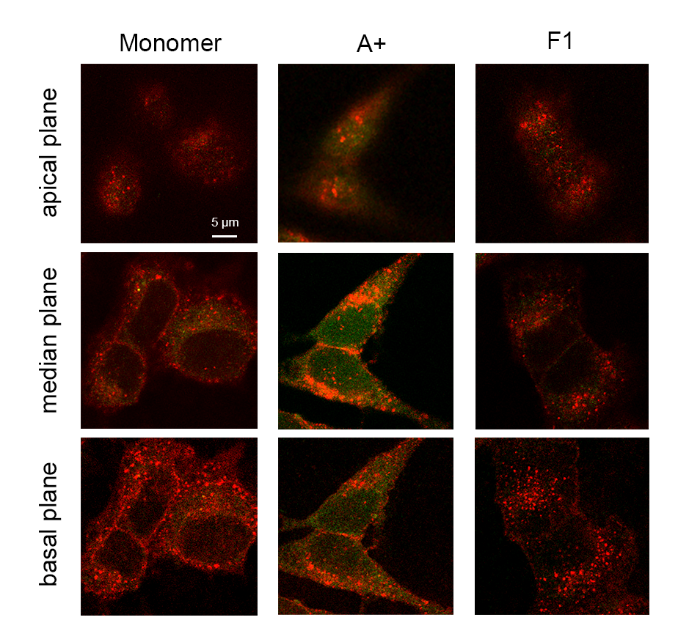


**Figure S2. DesAb-O detects Aβ_42_ oligomers bound to the neuronal membrane and internalized into the cytosol.** Representative STED microscopy images showing the basal, median, and apical sections of SH-SY5Y cells treated for 1 h with the indicated Aβ_42_ species at 3.0 μM (monomer equivalents) for 1 h. Red and green fluorescence indicates respectively the cell membranes and the Aβ_42_ species, detected with wheat germ agglutinin (WGA) and DesAb-O Ab.


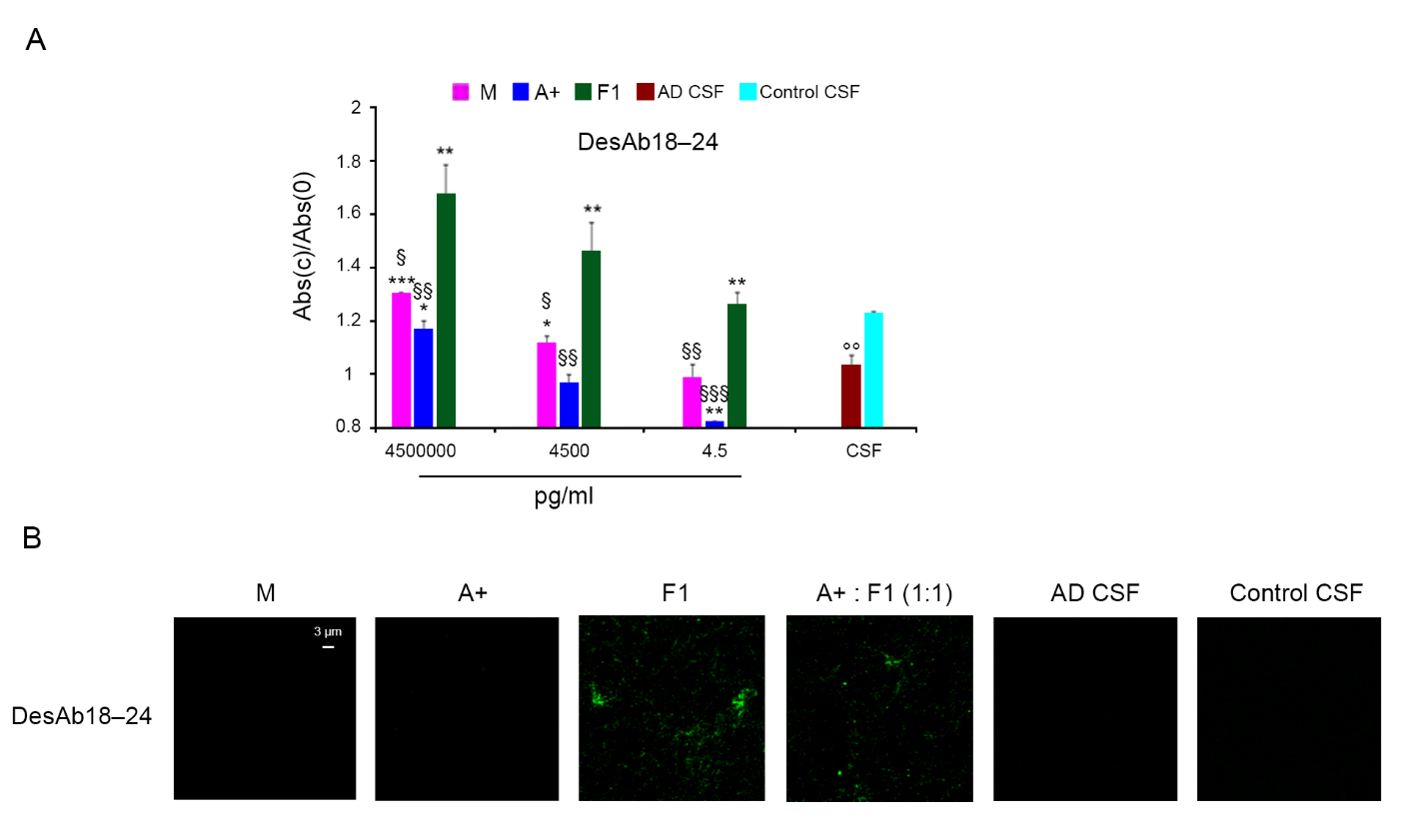

**Figure S3. DesAb18–24 detects Aβ_42_ fibrils and shows a non-specific signal in the CSF samples** (**A**) Sandwich ELISA assay. 0.25 mg/ml of CSF samples from AD patients and control subjects were adsorbed and quantified using DesAb18–24 at 0.5 µM. Standard curve was obtained with decreasing concentration of Aβ_42_ species formed *in vitro*. Data were normalized for the corresponding average value at concentration 0 pg/ml). Experimental errors are S.E.M. (n=4). Samples were analysed by Student *t* test relative to 0 pg/ml (* P<0.05, **P<0.01, *** P<0.001) or to F1 (§ P<0.05, §§ P<0.01, §§§P<0.001) or to control CSF (°° P<0.01). (**B**) Representative STED images showing Aβ_42_ species (M, A+ oligomers, F1, and a mixture containing both A+ and F1 at 1:1 molar ratio) and CSFs collected from AD patients and controls (n=4) spotted in a glass coverslip at 25 µM and 0.5 mg/ml, respectively. The green fluorescent signals arise from the staining with DesAb18–24.
